# Supplementary material for: Fetuin-A is a HIF target that safeguards tissue integrity during hypoxic stress
Source: Nat Commun. 2021 Jan 22;12:549. doi: 10.1038/s41467-020-20832-7 (PMC7822914; doi:10.1038/s41467-020-20832-7)
Supplement: Supplementary file 1 — Supplementary Information [file 41467_2020_20832_MOESM1_ESM.pdf]

Supplementary information for

Fetuin-A is a HIF target that safeguards tissue integrity during hypoxic stress

Authors and affiliations:

Stefan Rudloff[1,2], Mathilde Janot[1,2], Stephane Rodriguez[1,2,4], Kevin Dessalle[1,2], Willi Jahnen-Dechent[3] and Uyen Huynh-Do[1,2*]

[1] Department of Nephrology and Hypertension, Bern University Hospital, Freiburgstrasse 15, CH-3010 Bern, Switzerland

[2] Department of Biomedical Research, University of Bern, Freiburgstrasse 15, CH-3010 Bern, Switzerland

[3] Helmholtz-Institute for Biomedical Engineering, Biointerface Laboratory, RWTH Aachen University Medical Faculty, Pauwelsstrasse 30, 52074 Aachen, Germany

[4Present address:] Department of Onco-haematology, Geneva Medical University, Geneva, Switzerland

*corresponding author: uyen.huynh-do@insel.ch

Supplementary Figure 1:

Chronic fetal hypoxia induces intra-uterine growth restriction in mice

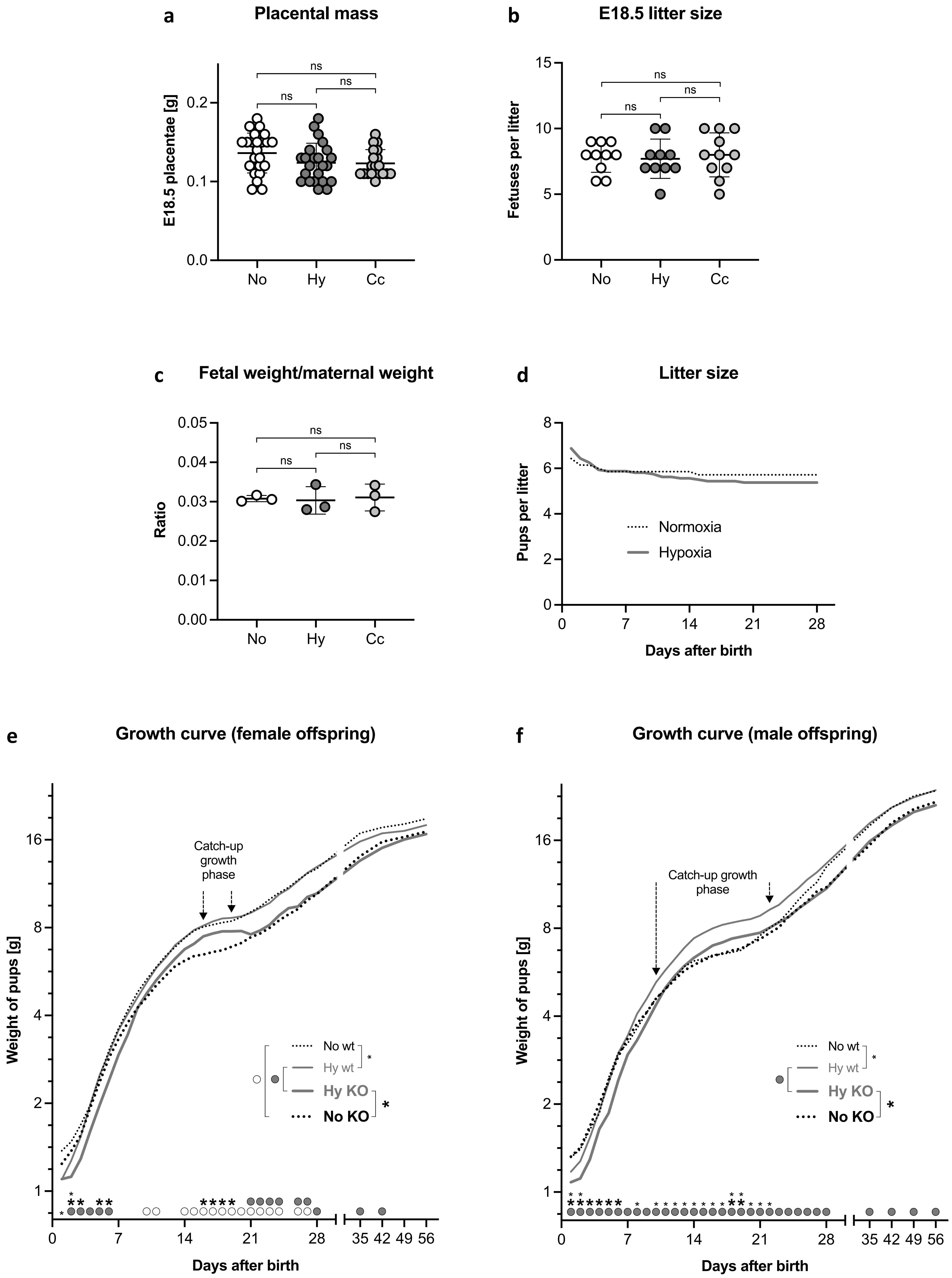

Supplementary Figure 2:

Kidneys of IUGR fetuses have fewer nephrons

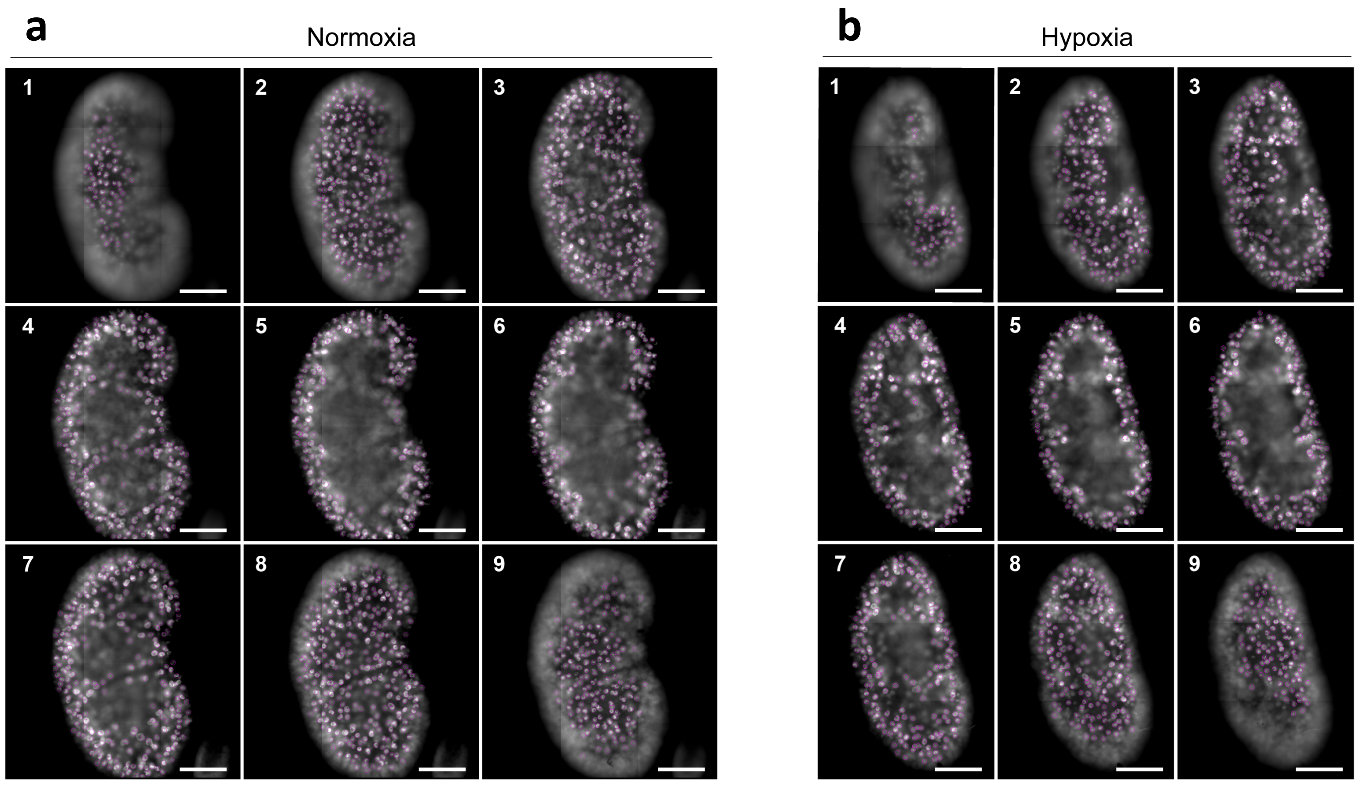

Supplementary Figure 3:

Hypoxia-induced gene expression in the kidney

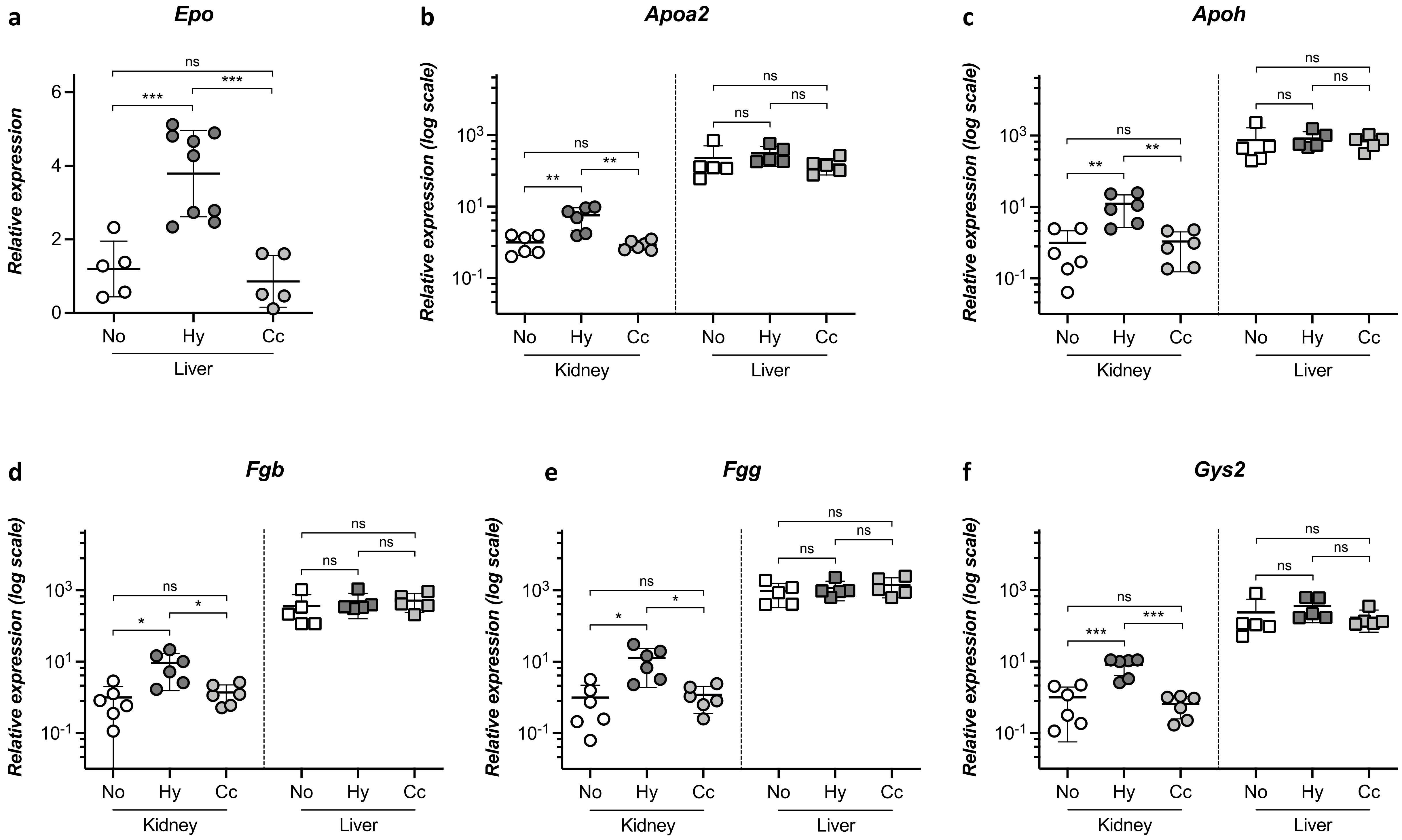

Supplementary Figure 4:

Localization of fetuin-A transcripts and protein in fetal kidneys

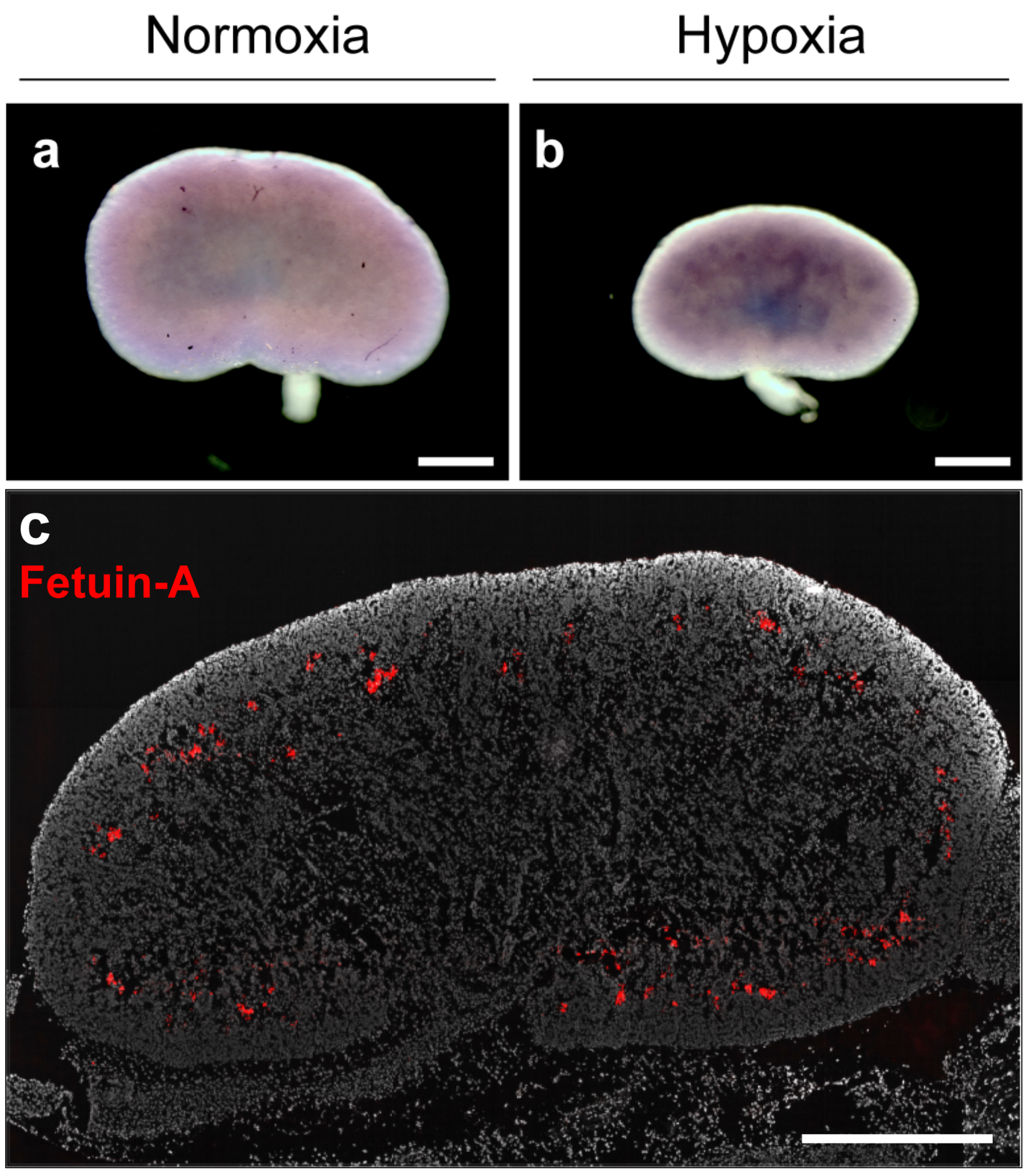

Supplementary Figure 5:

Identification of putative HIF binding sites of the fetuin-A gene locus

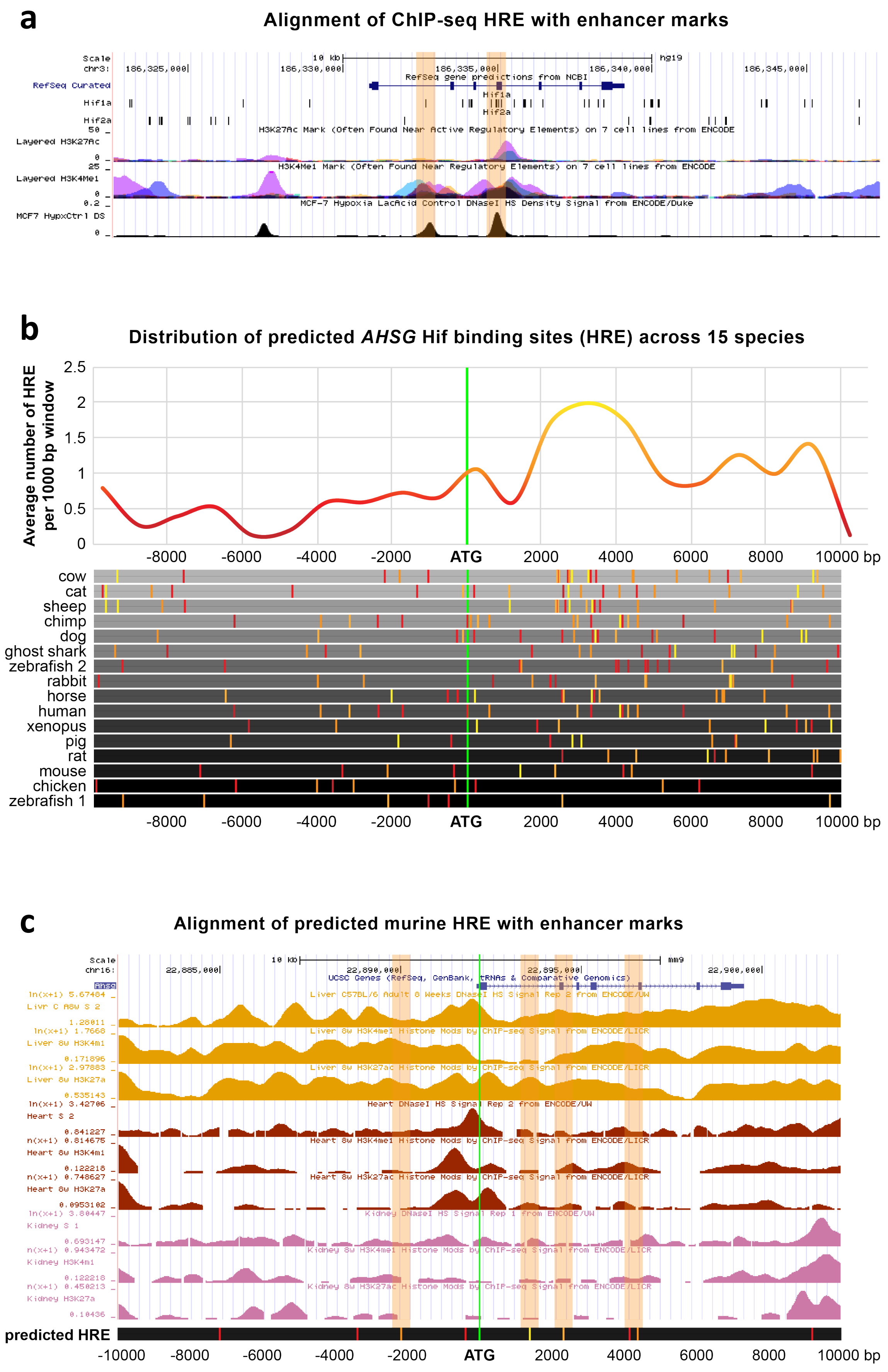

Supplementary Figure 6:

Increased expression of fetuin-A and fibrotic markers in hypoxia

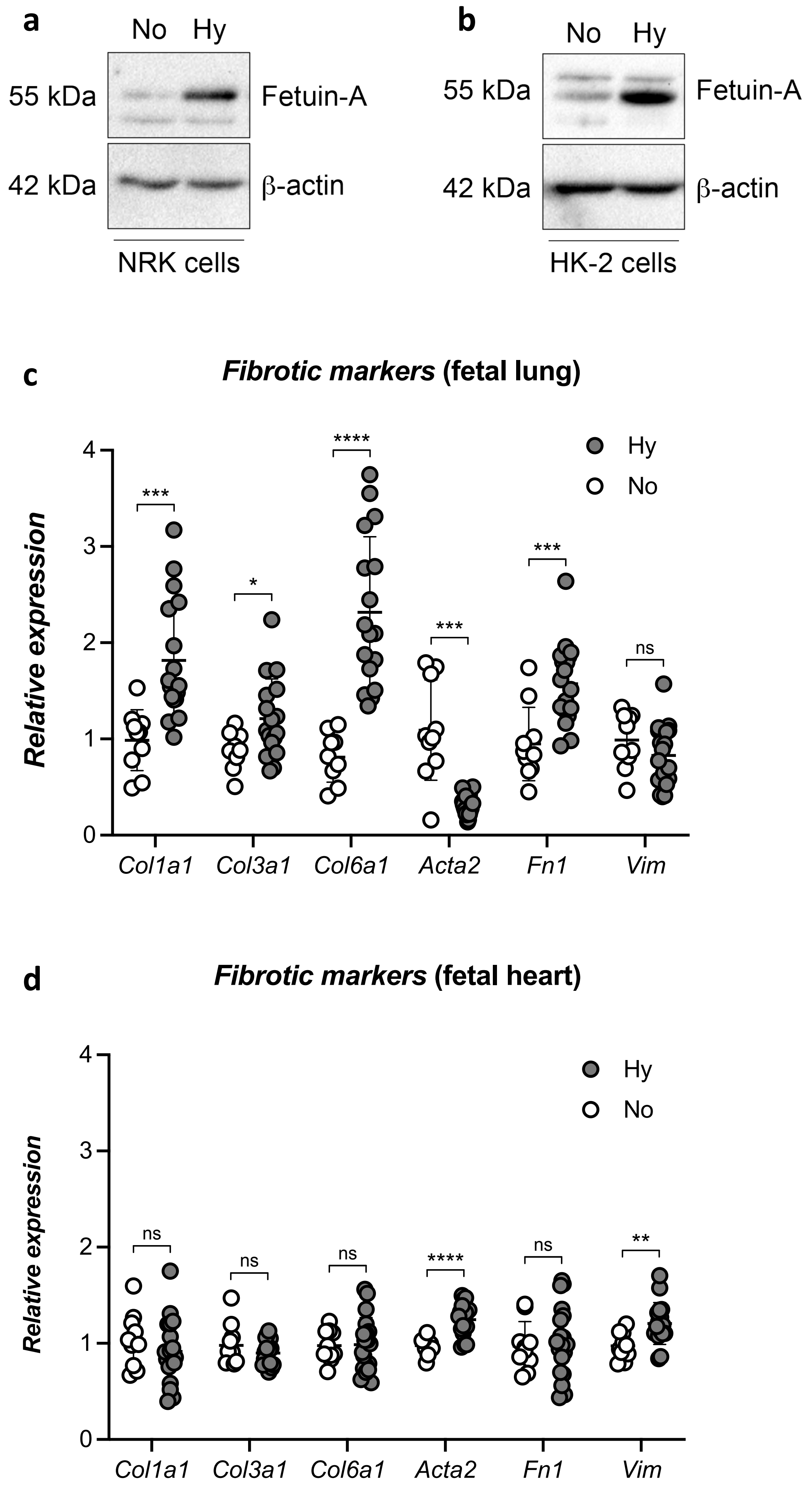

Supplementary Figure 7:

Increased expression of fibrosis markers in hypoxic *Ahsg* KO kidneys

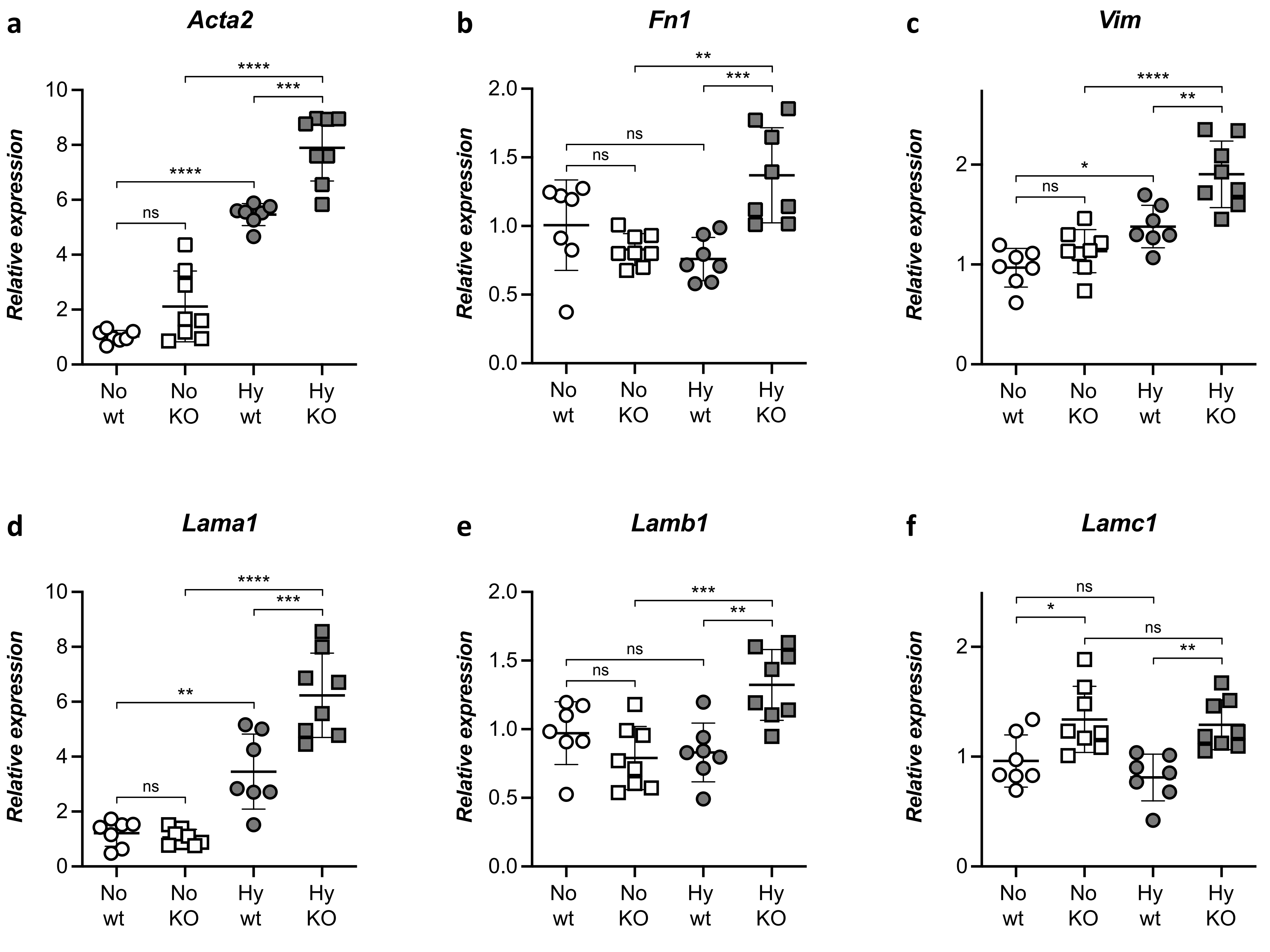

Supplementary Figure 8:

Fetuin-A attenuates hypoxia-induced expression of fibrotic markers

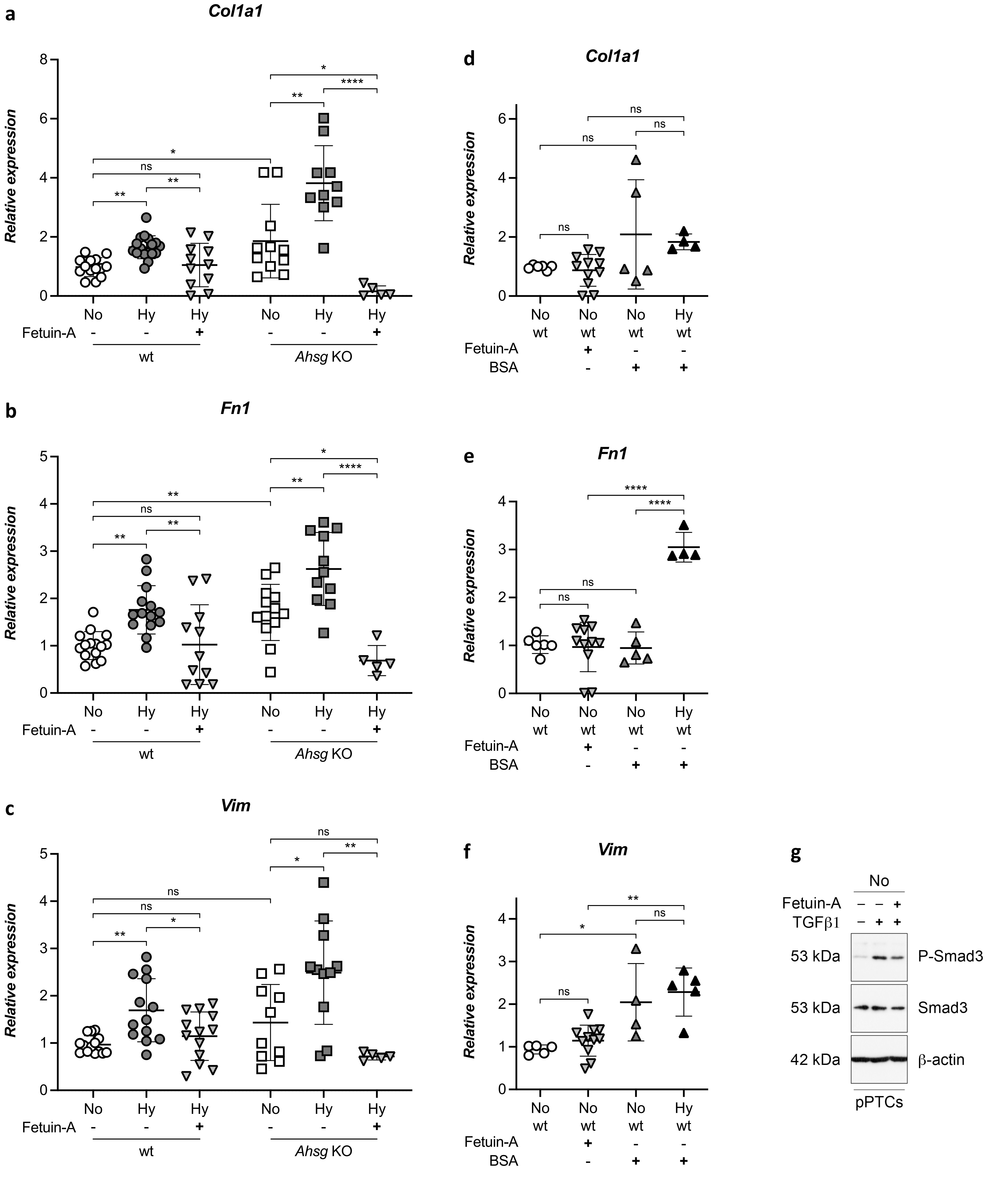

Supplementary Figure 9:

Fetuin-A mitigates the polarization of pro-inflammatory M1 macrophages

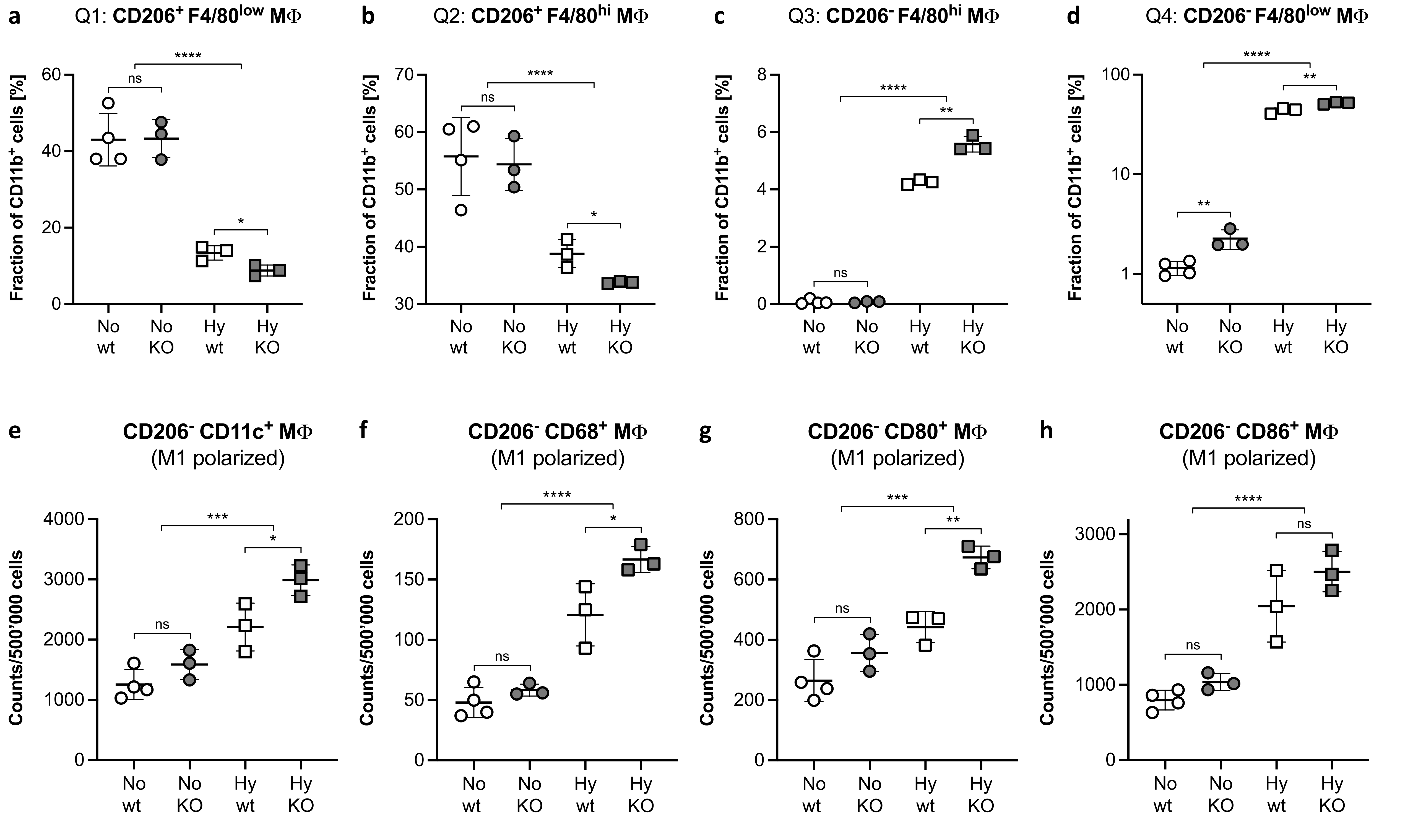

Supplementary Figure 10:

Apoptosis in hypoxic *Ahsg* KO kidneys

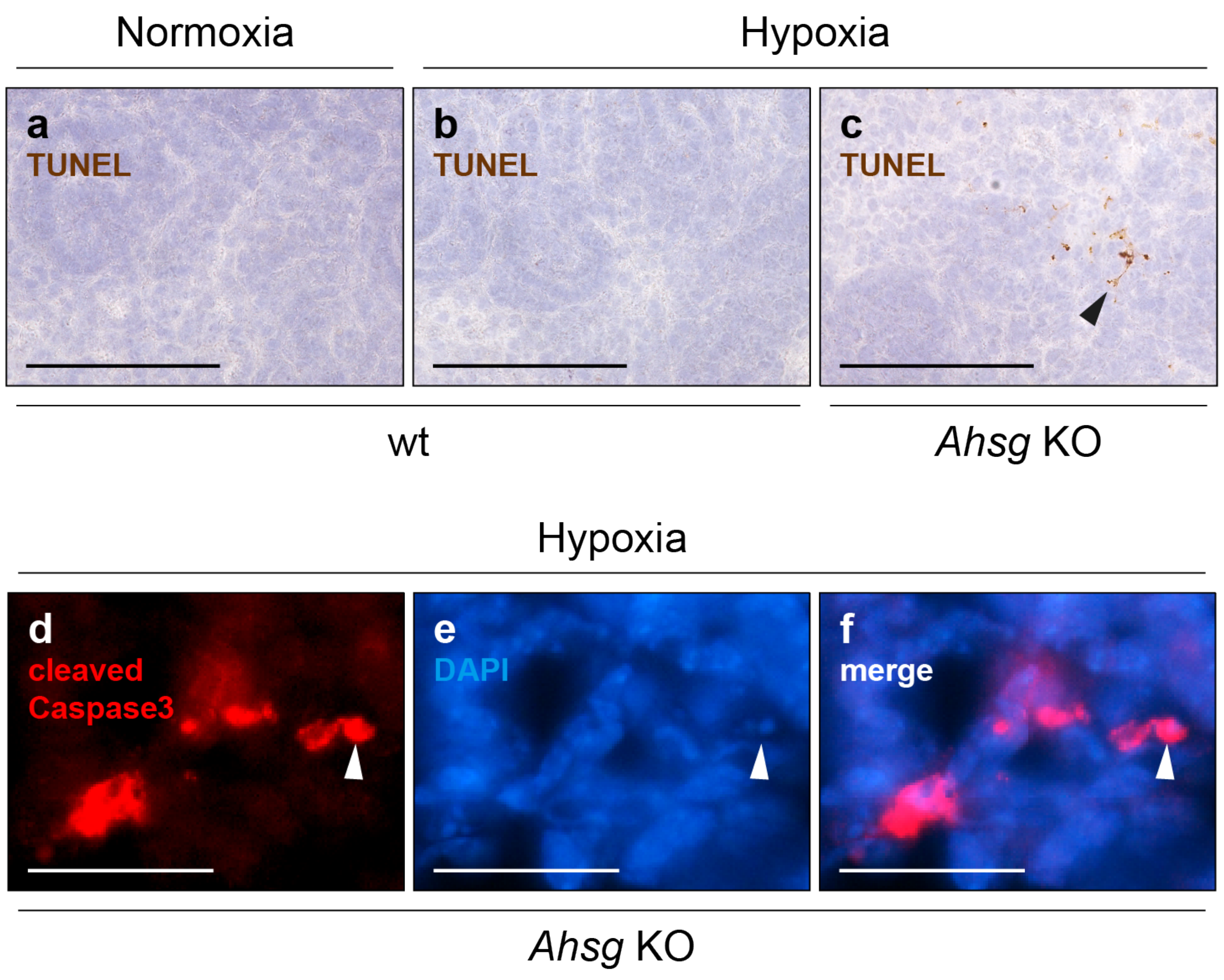

Supplementary Figure 11:

Translation of our IUGR model results into a clinical perspective

**Translation of renal insufficiency in our IUGR mouse model to CKD risk groups in human**				Proteinuria categories (mg/mmol Cr)<br>Description and sex-specific ranges					
				**P1**		**P2**		**P3**	
				Normal to mild increase		Mild to moderate increase		Severe increase	
				F: <90<br>M: <190		F: 90-120<br>M: 190-220		F: >120<br>M: >220	
GFR categories (µl/min/100g BW)<br>Description and range	**G1**	Normal to high	>900	**No wt F**	**No wt M**	**No KO F**	**Hy wt F**	**Hy wt M**	**No KO M**
	**G2**	Mild decrease	600-899					**Hy KO F**	**Hy KO M**
	**G3a**	Mild to moderate decrease	450-599						
	**G3b**	Moderate to severe decrease	300-449						
	**G4**	Severe decrease	150-299						
	**G5**	Kidney failure	<150						

KDIGO 2012 risk classifaction	low risk	moderate risk	high risk	very high risk

Supplementary Figure 12:

FACS gating strategy

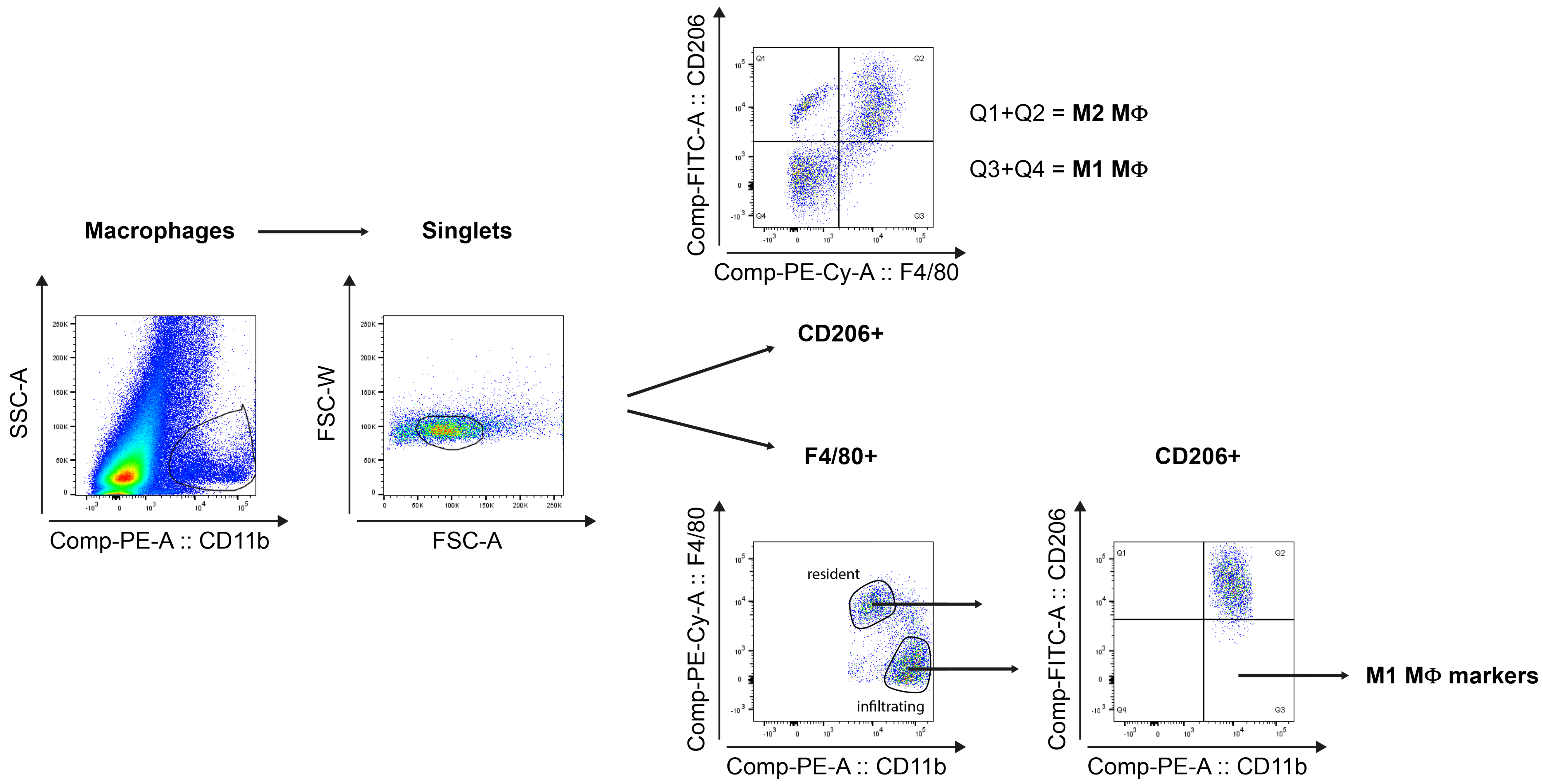

Supplementary Figure legends

Supplementary Figure 1 Chronic fetal hypoxia induces intra-uterine growth restriction in mice. a Weight of E18.5 placentae (n = 24 No, 23 Hy, and 19 Cc placentae). **b** Number of pups per litter (n = 10 No, 10 Hy, and 11 Cc litters). **c** E18.5 fetal/maternal weight ratio (n = 3 ratios for each condition). **d** Litter size throughout the suckling phase did not differ for normoxic and hypoxic pups. **e**,**f** Postnatal growth of female (**e**) and male offspring (**f**). Hypoxic offspring (*Ahsg* KO – bold grey line, wild-type – thin grey line) and normoxic offspring (*Ahsg* KO – bold black dotted line, wild-type – thin black dotted line). KOs weighed less than wild-types, catch-up growth was observed for hypoxic offspring during the third week after birth. Grey circles denote significance ($P < 0.05$) between hypoxic offspring, white circles between normoxic offspring, large asterisks between KOs, and small asterisks between wild-types. Data are presented as mean ± SEM (**a**-**c**) or mean only (**d**-**f**). Ordinary one-way ANOVA with Tukey's multiple comparison test (**a**-**c**), Multiple 2-sided t tests **(d-f)**. (ns = $P > 0.05$). Source data are provided as a Source Data file.

Supplementary Figure 2 Kidneys of IUGR fetuses have fewer nephrons. a,**b** 9 Z-stack images of a tissue cleared E18.5 normoxic (**a**) or hypoxic (**b**) kidney using the same magnification, taken at 100µm distance. Purple circles enclose glomeruli stained for nephrin. Images are representative of at least 6 iDisco-stained kidneys. Scale bar = 500 µm.

Supplementary Figure 3 Hypoxia-induced gene expression in the kidney. a Relative *Epo* mRNA levels in E18.5 fetal livers (n = 5 No, 9 Hy, and 5 Cc fetal livers). **b**-**f** Relative mRNA values of *Apoa2* (**b**), *Apoh* (**c**), *Fgb* (**d**), *Fgb* (**e**) and *Gys*2 (**f**) in E18.5 kidneys (circles) or

liver samples (squares). N = 6 kidneys for each condition or 5 livers for each condition. Kidney and liver samples are analyzed separately. Note the logarithmic scale on the Y-axis. Data are presented as mean ± SEM (**a**-**f**) or mean only (**d**-**f**). Ordinary one-way ANOVA with Tukey's multiple comparison test (**a**-**f**). (*** P < 0.001; ** P < 0.01; * P < 0.05; ns = P > 0.05). Source data are provided as a Source Data file.

Supplementary Figure 4 Localization of fetuin-A transcript and protein in fetal kidneys. **a**,**b** *In situ* hybridization for *Ahsg* in normoxic (**a**) or hypoxic (**b**) E18.5 whole-mount kidneys. **c** Immunofluorescent staining for Fetuin-A revealed that the protein was present in the outer renal cortex, just below the nephrogenic zone, forming an arch running along the circumference of the kidney, exactly where mature PTs are located in an E18.5 kidney. No staining was found in the inner cortex or the medulla. Images are representative of at least 3 independent experiments (**a**-**c**). Scale bar = 500 μm.

Supplementary Figure 5 Identification of putative HIF binding sites of the fetuin-A locus. **a** Genome browser image of human *AHSG* (top) illustrating the proximity of putative HIF binding sites (hypoxia response element – HRE) with epigenetic markers of active regulatory elements (H3K27Ac and H3K4Me1, middle) and open chromatin (DNaseI hypersensitivity, bottom). The orange bars traversing the image highlight two HREs overlapping with these epigenetic markers. **b** Location of potential HREs for fetuin-A of 15 different species. The upper panel shows the average distribution of these sites per 1kb window relative to the ATG (green line) of the fetuin-A genes. The lower panel depicts the exact locations of the predicted HREs for each species. The species are sorted (from top to bottom) according to the number of predicted HREs, Zebrafish has two *Ahsg* genes. HREs

with a relative score >0.9 are shown in red, >0.93 in orange and >0.97 in yellow. **c** Genome browser image of mouse *Ahsg* (top) illustrating the proximity of predicted HREs with H3K27Ac, H3K4Me1 and DNaseI hypersensitivity in heart (brown), kidney (purple) or liver (orange). The orange bars traversing the image highlight HREs with a relative score >0.93.

Supplementary Figure 6 Increased expression of fetuin-A and fibrotic markers in hypoxia. **a**,**b** Expression of fetuin-A protein in the rat cell line NRK (**a**) or in the human cell line HK-2 (**b**) cultured under normoxic or hypoxic conditions. Images are representative of two independent Western blots. Uncropped blots in Source Data. **c**,**d** Relative mRNA expression levels of collagens (*Col1a1*, *Col3a1*, and *Col6a1*), α-smooth muscle actin (*Acta2*), fibronectin (*Fn1*), and vimentin (*Vim*) in lungs (**c**) or hearts (**d**) from normoxic (white circles) or hypoxic fetuses (grey circles). Data were analyzed from n = 9-10 No and 17-18 Hy fetal lungs, or 10-11 No and 17-19 Hy fetal hearts and are shown as mean ± SEM. Unpaired 2-sided t test (with Welch's correction for lung *Col1a1*, *Col3a1*, *Col6a*, and Acta2). (**** P < 0.0001; *** P < 0.001; ** P < 0.01; * P < 0.05; ns = P > 0.05). Source data are provided as a Source Data file.

Supplementary Figure 7 Increased expression of fibrosis markers in kidneys of hypoxic *Ahsg* KO adult offspring. **a**-**f** Relative mRNA expression levels of *Acta2* (**a**), *Fn1* (**b**), *Vim* (**c**), *Lama1* (**d**), *Lamb1* (**e**), and *Lamc1* (**f**) were markedly enhanced in kidneys of hypoxic *Ahsg* KO offspring. Data were analyzed from n = 7 No wt, 8 No KO, 7 Hy wt, and 8 Hy KO offspring and are presented as mean ± SEM. Ordinary one-way ANOVA with Tukey's multiple comparisons test. (**** P < 0.0001; *** P < 0.001; ** P < 0.01; * P < 0.05; ns = P > 0.05). Source data are provided as a Source Data file.

Supplementary Figure 8 Fetuin-A attenuates hypoxia-induced expression of fibrotic markers *in vitro*. a-c Fetuin-A supplementation (downward pointing triangles) attenuated the hypoxia-induced mRNA expression of *Col1a1* (**a**), *Fn1* (**b**), and *Vim* (**c**) in pPTCs. N = 14-15 No wt, 13-17 Hy wt, 10-13 No *Ahsg* KO and 10-11 Hy *Ahsg* KO samples without fetuin-A supplementation, and 11-13 Hy wt and 5 Hy *Ahsg* KO samples with fetuin-A supplementation. Wt and *Ahsg* KO samples are analyzed separately. Unpaired 2-tailed t test with Welch's correction (only for comparison of normoxic wt and normoxic *Ahsg* KO samples). **d-f** BSA supplementation (upward pointing triangles), did not reduce the expression of *Col1a1* (**d**), *Fn1* (**e**), and *Vim* (**f**) in wt pPTCs. N = 5-6 No samples without treatment, 11 No samples with fetuin-A treatment, 4-5 No and 4-5 Hy samples with BSA treatment. **g** Simultaneous treatment with TGF-β1 and fetuin-A reduced the phosphorylation of Smad3 in pPTCs. Images are representative of 2 Western blots. Uncropped blots in Source Data. Data were analyzed from n = pPTCs derived from kidneys of wt or *Ahsg* KO mice and are presented as mean ± SEM (**a-f**). Ordinary one-way ANOVA with Tukey's multiple comparisons test (**a-f**). (**** $P < 0.0001$; ** $P < 0.01$; * $P < 0.05$; ns = $P > 0.05$). Source data are provided as a Source Data file.

Supplementary Figure 9 Fetuin-A mitigates the polarization of pro-inflammatory M1 macrophages in fetal hypoxic kidneys. a-d Quantification of the 4 quadrants of the FACS blots shown in Figs. 7g-j. **e-h** Graphical presentation of the data shown in Table 1. Data were analyzed from n = 4 No wt, 3 No KO, 3 Hy wt, and 3 Hy KO fetal kidneys and are presented as mean ± SEM (**a-h**). Unpaired 2-tailed t test (with Welch's correction if required) (**a-h**). (**** $P < 0.0001$; *** $P < 0.001$; ** $P < 0.01$; * $P < 0.05$; ns = $P > 0.05$). Source data are provided as a Source Data file.

Supplementary Figure 10 Apoptosis in hypoxic *Ahsg* KO kidneys. a-c TUNEL staining on E18.5 kidney sections of normoxic wt (**a**), hypoxic wt (**b**) or hypoxic *Ahsg* KO mice (**c**). The black arrowhead points towards TUNEL positive cells. **d-f** Cleaved caspase-3 on E18.5 kidney sections of hypoxic *Ahsg* KO mice. The white arrowhead highlights a cell with a fragmented nucleus. Images are representative of at least 3 independent experiments. Scale bar = 100 µm (**a-c**) or 50 µm (**d-f**).

Supplementary Figure 11 Translation of our IUGR mouse model results into a clinical perspective, using KDIGO 2012 classification of CKD. Mouse data is written in bold. Wt mice (males and females) have no or only a low risk for CKD (green). Normoxic *Ahsg* KO females and hypoxic wt females have a moderate risk for disease (yellow), whereas all remaining males and hypoxic *Ahsg* KO females are staged into a high risk category (orange). F, female; M, male; No, normoxic; Hy, hypoxic; wt, wild type; KO, Ahsg KO.

Supplementary Figure 12 FACS gating strategy. Strategy used to gate the cells.

Supplementary Tables

Supplementary Table 1 Induced genes (Hypoxia vs. Control groups).

Gene symbol	Fold change	Induced by hypoxia	CPP component
Ahsg	9.12		**x**[1, 2]
Fgg	5.84	Vij, 2009[3]	
Alb	5.21	Suresh, 2018[4]	**x**[1, 2]
Apoh	5.18		
Hbb-y	4.86		
Fgb	4.21		
Trf	3.95	Rolfs, 1997[5]	**x**[1, 2]
Pzp	3.93		
Apoa2	3.79		**x**[1, 2]
Apoa1	3.68	Padhy, 2013[6]	**x**[1, 2]
Kng1	3.48		**x**[2]
Angptl3	2.97		
Plg	2.91		**x**[2]
F2	2.87	Tripathy, 2013[7]	**x**[1, 2]
Serpind1	2.64		
Cps1	2.60		
Rbp4	2.58		
Fga	2.56	Vij, 2009[3]	**x**[52]
Itih3	2.50		
Apoc2	2.48		
Gys2	2.46		
Apob	2.45		
Serpinc1	2.41		**x**[1, 2]
Itih2	2.39		
Hpd	2.30		
Serpina1d	2.22	Wenger, 1995[8]	**x**[1, 2]
Hpx	2.20	Tan, 2016[9]	
A2m	2.18	Zhang, 2017[10]	**x**[1, 2]
C8b	2.13		
Nrn1	2.03	Le Jan, 2006[11]	
Serpina3n	2.00	Wenger, 1995[8]	
Itih4	1.97		
Hba-x	1.96		
Car1	1.96		
Apof	1.94		
Uox	1.84		
Apoc4	1.75		
Rgn	1.75		
Cirbp	1.74	Wellmann, 2004[12]	
Apoc1	1.70		
Crp	1.70		
Lect2	1.69		
Scd1	1.69	Zhang, 2014[13]	
Pemt	1.57		
Aox3	1.52		
Lcat	1.50		
Vstm2b	1.45		

Lss	1.45		
Tff3	1.41	Hernández, 2009[14]	
St6gal2	1.41		
Apoa4	1.40		**x**[2]
Hal	1.38		
F12	1.38	Liangos, 2010[15]	
Akr1c18	1.38	Saraf, 2018[16]	
Marcksl1	1.37		
Sc4mol	1.36		
Hc	1.36	Kelly, 2015[17]	
Tubb4	1.35		
Zfp395	1.33		
Hmgcr	1.33		
Itih1	1.32		
Txnrd3	1.31		

Supplementary Table 2 Repressed genes (Hypoxia vs. Control groups).

**Gene symbol**	**Fold change**
Napsa	-1.71
Fmo2	-1.68
Ccl3	-1.64
Acot11	-1.59
Pappa2	-1.59
Slc10a5	-1.55
Ugt2b37	-1.54
Pof1b	-1.53
Cntn4	-1.53
Lrrc19	-1.51
Glipr1	-1.49
Gcet2	-1.49
Gbp2	-1.44
C1ra	-1.44
Kcnj15	-1.42
Cxcl13	-1.40
Emr1	-1.40
Asah2	-1.40
Zfp760	-1.38
Scn7a	-1.38
Xpnpep2	-1.37
Prkaa2	-1.36
Pfn3	-1.35
Dpp7	-1.34
Slc25a45	-1.34
Sord	-1.34
Hsph1	-1.33
Pdk4	-1.32

Supplementary Table 3 Functional annotation using standard settings provided by DAVID.

induced genes (62)					
**Category**	**Term**	**Count**	**%**	**P-Value**	**Fold Enrichment**
UP_KEYWORDS	Secreted	35*	57.4%	2.90E-23	7.7
UP_TISSUE	Plasma	16*	26.2%	1.40E-22	57.0
UP_TISSUE	Liver	43*	70.5%	6.80E-18	3.7
KEGG_PATHWAY	Complement and coagulation cascades	13	21.3%	3.40E-16	35.7
GOTERM_MF_ALL	Endopeptidase inhibitor activity	13*	21.3%	2.00E-12	19.3
GOTERM_BP_ALL	Acute inflammatory response	10*	16.4%	3.60E-10	23.0
UP_KEYWORDS	Lipid transport	7	11.5%	9.90E-08	30.6
GOTERM_BP_ALL	Acute-phase response	6*	9.8%	4.30E-07	38.0
UP_KEYWORDS	Lipid metabolism	10	16.4%	1.40E-06	8.9
GOTERM_BP_ALL	Regulation of response to stress	16*	26.2%	4.40E-06	4.0
repressed genes (28)					
**Category**	**Term**	**Count**	**%**	**P-Value**	**Fold Enrichment**
GOTERM_BP_ALL	Lymphocyte migration	3	10.7	5.50E-03	25.8
GOTERM_MF_ALL	Peptidase activity	5	17.9	1.70E-02	4.8
UP_TISSUE	Kidney	9	32.1	2.30E-02	2.4

Supplementary Table 4 qPCR assays and primers.

TaqMan Gene Expression Assays (ThermoFisher)	
**Gene Symbol**	**AssayID**
18s	Mm03928990_g1
Ahsg	Mm01145470_m1
Apoa2	Mm00442687_m1
Apoc2	Mm00437571_m1
Apoh	Mm00496516_m1
Fgb	Mm00805336_m1
Fgg	Mm00513575_m1
Gys2	Mm01267381_g1

Universal Probe Library (Roche)			
**Gene Symbol**	**Forward primer**	**Reverse Primer**	**Probe Number**
Acta2	actctcttccagccatctttca	ataggtggtttcgtggatgc	58
Actb	ctaaggccaaccgtgaaaag	accagaggcatacagggaca	64
Col1a1	aggcaagcctggtgaaca	accagggaaacctctctcg	80
Col3a1	tggaccccaaggtcttcc	catctgatccagggtttcca	64
Col6a1	accggttgagcaaggatg	tccacgtgctcttgcatct	5
Fn1	tgccctgaagaacaatcaga	aaccagttggggaagctcat	69
Ppia	acgccactgtcgcttttc	ctgcaaacagctcgaagga	46
Lama1	cccgacaacctcctcttcta	catctccactgcgagaaagtc	74
Tgfb1	tggagcaacatgtggaactc	gtcagcagccggttacca	72
Vim	gtaccggagacaggtgcagt	ttctcttccatctcacgcatc	1

Supplementary Table 5 Location of putative HREs for the fetuin-A genes of 15 species. HREs with a relative score >0.9 are shown in red, >0.93 in orange and >0.97 in yellow.

Cat					
Site	Score	Rel. score	Start	Strand	Sequence
1	8.275	0.91277	-9791	-1	GAACGTGA
2	9.739	0.95644	-9703	1	GCACGTGG
3	10.232	0.97115	-9703	-1	CCACGTGC
4	8.984	0.93392	-8483	-1	CCACGTGG
5	8.984	0.93392	-8483	1	CCACGTGG
6	8.295	0.91337	-7932	1	GGGCGTGG
7	8.850	0.92992	-4713	1	CCACGTGA
8	8.409	0.91677	-1376	1	GAACGTGG
9	9.951	0.96277	-148	-1	GGACGTGG
10	8.409	0.91677	150	-1	GAACGTGG
11	9.605	0.95245	1086	-1	GCACGTGA
12	8.161	0.90937	2537	1	GGGCGTGA
13	9.739	0.95644	2671	-1	GCACGTGG
14	10.232	0.97115	2671	1	CCACGTGC
15	9.148	0.93881	3007	-1	AGACGTGG
16	8.664	0.92437	3604	-1	CTGCGTGC
17	9.148	0.93881	4038	-1	AGACGTGG
18	8.850	0.92992	4502	-1	CCACGTGA
19	9.148	0.93881	4979	-1	AGACGTGG
20	8.984	0.93392	6976	-1	CCACGTGG
21	8.984	0.93392	6976	1	CCACGTGG
22	10.987	0.99367	8812	-1	GCACGTGC
23	10.987	0.99367	8812	1	GCACGTGC
24	9.196	0.94024	9487	1	CGACGTGG

Chicken					
Site	Score	Rel. score	Start	Strand	Sequence
1	8.616	0.92294	-9959	-1	ATGCGTGC
2	8.711	0.92578	-6227	-1	CTACGTGT
3	9.148	0.93881	-4055	1	AGACGTGG
4	8.047	0.90597	-3633	1	GAACGTGT
5	9.014	0.93482	-3076	1	AGACGTGA
6	8.854	0.93004	-363	-1	AAACGTGC
7	8.575	0.92172	189	1	CCGCGTGC
8	8.984	0.93392	5197	-1	CCACGTGG
9	8.984	0.93392	5197	1	CCACGTGG
10	8.663	0.92434	6173	-1	ATACGTGT

Chimp					
Site	Score	Rel. score	Start	Strand	Sequence
1	8.037	0.90567	-6262	1	GTGCGTGA
2	9.148	0.93881	-3952	-1	AGACGTGG
3	9.605	0.95245	-3178	1	GCACGTGA
4	8.295	0.91337	-2429	-1	GGGCGTGG
5	8.295	0.91337	-1774	-1	GGGCGTGG
6	8.740	0.92664	-30	-1	AGGCGTGC
7	8.984	0.93392	58	-1	CCACGTGG
8	8.984	0.93392	58	1	CCACGTGG

Human					
Site	Score	Rel. score	Start	Strand	Sequence
1	8.037	0.90567	-6273	1	GTGCGTGA
2	9.148	0.93881	-3959	-1	AGACGTGG
3	9.605	0.95245	-3186	1	GCACGTGA
4	8.295	0.91337	-2413	-1	GGGCGTGG
5	8.295	0.91337	-1762	-1	GGGCGTGG
6	8.740	0.92664	-30	-1	AGGCGTGC
7	8.891	0.93115	556	-1	ATACGTGA
8	9.951	0.96277	2912	1	GGACGTGG
9	8.850	0.92992	3280	-1	CCACGTGA
10	11.199	1.00000	4056	1	GGACGTGC
11	8.788	0.92807	4119	-1	CGGCGTGC
12	9.828	0.95910	4269	1	GTACGTGG
13	9.196	0.94024	4526	-1	CGACGTGG
14	8.161	0.90937	5749	-1	GGGCGTGA
15	9.419	0.94690	8512	1	GTGCGTGC
16	9.014	0.93482	9651	1	AGACGTGA

Mouse					
Site	Score	Rel. score	Start	Strand	Sequence
1	8.575	0.92172	-7181	1	CCGCGTGC
2	8.409	0.91677	-3376	1	GAACGTGG
3	9.196	0.94024	-2167	-1	CGACGTGG
4	8.850	0.92992	-390	-1	CCACGTGA
5	10.321	0.97381	1386	1	CTACGTGC
6	9.605	0.95245	2318	1	GCACGTGA
7	8.711	0.92578	4143	-1	CTACGTGT
8	8.936	0.93249	4368	-1	ACACGTGG
9	8.623	0.92315	4368	1	CCACGTGT
10	8.575	0.92172	9190	-1	ACACGTGT
11	8.575	0.92172	9190	1	ACACGTGT

Pig					
Site	Score	Rel. score	Start	Strand	Sequence
1	9.466	0.94830	-6362	-1	GTACGTGT
2	9.739	0.95644	-1879	1	GCACGTGG
3	10.232	0.97115	-1879	-1	CCACGTGC
4	8.788	0.92807	-464	1	CGGCGTGC
5	8.295	0.91337	2185	-1	GGGCGTGG
6	9.739	0.95644	2780	-1	GCACGTGG
7	10.232	0.97115	2780	1	CCACGTGC
8	10.321	0.97381	3020	1	CTACGTGC
9	8.984	0.93392	6520	-1	CCACGTGG
10	8.984	0.93392	6520	1	CCACGTGG
11	8.275	0.91277	7140	1	GAACGTGA
12	8.854	0.93004	7165	-1	AAACGTGC

Rabbit					
Site	Score	Rel. score	Start	Strand	Sequence

9	9.828	0.95910	250	1	GTACGTGG
10	8.891	0.93115	556	-1	ATACGTGA
11	8.902	0.93147	2810	-1	CAACGTGC
12	9.951	0.96277	2916	1	GGACGTGG
13	8.850	0.92992	3281	-1	CCACGTGA
14	11.199	1.00000	4057	1	GGACGTGC
15	8.788	0.92807	4120	-1	CGGCGTGC
16	9.828	0.95910	4270	1	GTACGTGG
17	9.196	0.94024	4527	-1	CGACGTGG
18	8.161	0.90937	5750	-1	GGGCGTGA
19	9.419	0.94690	8525	1	GTGCGTGC
20	9.014	0.93482	9665	1	AGACGTGA

Cow					
**Site**	**Score**	**Rel. score**	**Start**	**Strand**	**Sequence**
1	9.739	0.95644	-9399	-1	GCACGTGG
2	10.232	0.97115	-9399	1	CCACGTGC
3	8.275	0.91277	-7624	1	GAACGTGA
4	8.295	0.91337	-2242	1	GGGCGTGG
5	9.025	0.93514	-1847	-1	ATACGTGG
6	8.575	0.92172	-1077	1	CCGCGTGC
7	9.828	0.95910	2370	-1	GTACGTGG
8	8.295	0.91337	2384	-1	GGGCGTGG
9	9.605	0.95245	2424	1	GCACGTGA
10	8.295	0.91337	2656	-1	GGGCGTGG
11	9.014	0.93482	2694	1	AGACGTGA
12	9.739	0.95644	2768	-1	GCACGTGG
13	10.232	0.97115	2768	1	CCACGTGC
14	11.199	1.00000	3206	1	GGACGTGC
15	8.664	0.92437	3269	-1	CTGCGTGC
16	8.409	0.91677	3414	1	GAACGTGG
17	8.854	0.93004	4390	-1	AAACGTGC
18	8.939	0.93258	4417	1	CTACGTGA
19	8.902	0.93147	5570	-1	CAACGTGC
20	8.984	0.93392	6443	-1	CCACGTGG
21	8.984	0.93392	6443	1	CCACGTGG
22	8.802	0.92849	6941	-1	ACACGTGA
23	9.817	0.95877	7287	-1	GGACGTGA
24	10.987	0.99367	9222	-1	GCACGTGC
25	10.987	0.99367	9222	1	GCACGTGC
26	9.657	0.95400	9333	-1	GAACGTGC

Dog					
**Site**	**Score**	**Rel. score**	**Start**	**Strand**	**Sequence**
1	9.196	0.94024	-8321	1	CGACGTGG
2	9.951	0.96277	-4021	-1	GGACGTGG
3	8.409	0.91677	-310	-1	GAACGTGG
4	9.951	0.96277	-149	-1	GGACGTGG
5	8.409	0.91677	150	-1	GAACGTGG
6	8.835	0.92948	1391	1	CGACGTGT
7	8.295	0.91337	2508	-1	GGGCGTGG
8	9.148	0.93881	2824	-1	AGACGTGG
9	8.295	0.91337	3321	1	GGGCGTGG

1	8.575	0.92172	-9898	1	CCGCGTGC
2	8.854	0.93004	-4033	-1	AAACGTGC
3	8.984	0.93392	-2804	-1	CCACGTGG
4	8.984	0.93392	-2804	1	CCACGTGG
5	8.788	0.92807	-1640	-1	CGGCGTGC
6	8.161	0.90937	653	-1	GGGCGTGA
7	8.939	0.93258	1709	1	CTACGTGA
8	8.295	0.91337	2190	-1	GGGCGTGG
9	8.171	0.90967	2327	-1	GTGCGTGG
10	9.951	0.96277	3402	-1	GGACGTGG
11	9.377	0.94564	4726	1	GCACGTGT
12	10.184	0.96972	4726	-1	ACACGTGC
13	9.073	0.93658	4754	1	CTACGTGG
14	9.657	0.95400	6992	1	GAACGTGC
15	9.739	0.95644	7020	1	GCACGTGG
16	10.232	0.97115	7020	-1	CCACGTGC
17	9.014	0.93482	7081	-1	AGACGTGA
18	8.575	0.92172	8660	-1	CCGCGTGC

Rat					
**Site**	**Score**	**Rel. score**	**Start**	**Strand**	**Sequence**
1	7.933	0.90257	2510	-1	GGGCGTGT
2	8.902	0.93147	3739	1	CAACGTGC
3	10.184	0.96972	4487	-1	ACACGTGC
4	9.377	0.94564	4487	1	GCACGTGT
5	10.396	0.97604	6401	1	AGACGTGC
6	8.000	0.90456	6587	-1	GAGCGTGC
7	9.073	0.93658	6887	-1	CTACGTGG
8	9.542	0.95057	8038	-1	GGGCGTGC
9	9.148	0.93881	9238	1	AGACGTGG
10	9.419	0.94690	9333	1	GTGCGTGC
11	9.014	0.93482	9955	-1	AGACGTGA

Sheep					
**Site**	**Score**	**Rel. score**	**Start**	**Strand**	**Sequence**
1	11.199	1.00000	-9703	1	GGACGTGC
2	9.739	0.95644	-9385	-1	GCACGTGG
3	10.232	0.97115	-9385	1	CCACGTGC
4	8.623	0.92315	-8196	-1	CCACGTGT
5	8.936	0.93249	-8196	1	ACACGTGG
6	8.275	0.91277	-7591	1	GAACGTGA
7	11.199	1.00000	1103	1	GGACGTGC
8	9.828	0.95910	2340	-1	GTACGTGG
9	8.295	0.91337	2354	-1	GGGCGTGG
10	9.605	0.95245	2394	1	GCACGTGA
11	8.295	0.91337	2591	-1	GGGCGTGG
12	9.739	0.95644	2703	-1	GCACGTGG
13	10.232	0.97115	2703	1	CCACGTGC
14	9.817	0.95877	3157	-1	GGACGTGA
15	11.199	1.00000	3311	1	GGACGTGC
16	8.788	0.92807	3374	-1	CGGCGTGC
17	8.409	0.91677	3519	1	GAACGTGG
18	8.939	0.93258	4530	1	CTACGTGA

10	11.199	1.00000	3397	1	GGACGTGC
11	8.788	0.92807	3460	-1	CGGCGTGC
12	9.951	0.96277	3944	-1	GGACGTGG
13	8.000	0.90456	4919	1	GAGCGTGC
14	9.025	0.93514	5038	-1	ATACGTGG
15	8.295	0.91337	6587	1	GGGCGTGG
16	11.199	1.00000	7863	1	GGACGTGC
17	10.396	0.97604	8913	-1	AGACGTGC
18	9.739	0.95644	9035	1	GCACGTGG
19	10.232	0.97115	9035	-1	CCACGTGC

Ghost shark					
**Site**	**Score**	**Rel. score**	**Start**	**Strand**	**Sequence**
1	9.062	0.93625	-9459	1	CGACGTGA
2	8.037	0.90567	-8059	-1	GTGCGTGA
3	8.623	0.92315	-4328	-1	CCACGTGT
4	8.936	0.93249	-4328	1	ACACGTGG
5	8.787	0.92804	-3823	1	AGACGTGT
6	9.377	0.94564	-2892	-1	GCACGTGT
7	10.184	0.96972	-2892	1	ACACGTGC
8	8.663	0.92434	1695	-1	ATACGTGT
9	9.014	0.93482	2970	1	AGACGTGA
10	9.466	0.94830	3273	-1	GTACGTGT
11	8.161	0.90937	4639	1	GGGCGTGA
12	8.275	0.91277	5381	-1	GAACGTGA
13	10.396	0.97604	5530	-1	AGACGTGC
14	10.396	0.97604	7044	-1	AGACGTGC
15	9.739	0.95644	7115	-1	GCACGTGG
16	10.232	0.97115	7115	1	CCACGTGC
17	8.047	0.90597	7684	1	GAACGTGT
18	9.062	0.93625	8196	-1	CGACGTGA
19	8.664	0.92437	9888	-1	CTGCGTGC

Horse					
**Site**	**Score**	**Rel. score**	**Start**	**Strand**	**Sequence**
1	9.605	0.95245	-6498	-1	GCACGTGA
2	11.199	1.00000	-2058	1	GGACGTGC
3	8.575	0.92172	-559	-1	ACACGTGT
4	8.575	0.92172	-559	1	ACACGTGT
5	8.409	0.91677	-293	-1	GAACGTGG
6	10.396	0.97604	171	-1	AGACGTGC
7	8.295	0.91337	2505	-1	GGGCGTGG
8	9.605	0.95245	2545	1	GCACGTGA
9	11.199	1.00000	3299	1	GGACGTGC
10	8.788	0.92807	3362	-1	CGGCGTGC
11	9.828	0.95910	3505	1	GTACGTGG
12	8.984	0.93392	6636	-1	CCACGTGG
13	8.984	0.93392	6636	1	CCACGTGG
14	9.148	0.93881	6792	-1	AGACGTGG
15	8.984	0.93392	6840	-1	CCACGTGG
16	8.984	0.93392	6840	1	CCACGTGG
17	9.073	0.93658	7917	-1	CTACGTGG

19	8.984	0.93392	6589	-1	CCACGTGG
20	8.984	0.93392	6589	1	CCACGTGG
21	8.787	0.92804	8640	1	AGACGTGT
22	9.377	0.94564	8662	-1	GCACGTGT
23	10.184	0.96972	8662	1	ACACGTGC

Xenopus					
**Site**	**Score**	**Rel. score**	**Start**	**Strand**	**Sequence**
1	8.171	0.90967	-5880	1	GTGCGTGG
2	9.466	0.94830	-3541	1	GTACGTGT
3	9.739	0.95644	223	1	GCACGTGG
4	10.232	0.97115	223	-1	CCACGTGC
5	8.711	0.92578	1837	-1	CTACGTGT
6	9.377	0.94564	2416	1	GCACGTGT
7	10.184	0.96972	2416	-1	ACACGTGC
8	9.419	0.94690	6455	1	GTGCGTGC
9	9.739	0.95644	7947	1	GCACGTGG
10	10.232	0.97115	7947	-1	CCACGTGC
11	8.850	0.92992	8780	-1	CCACGTGA
12	9.605	0.95245	9031	-1	GCACGTGA
13	8.711	0.92578	9180	1	CTACGTGT
14	10.396	0.97604	9707	-1	AGACGTGC

Zebrafish 1					
**Site**	**Score**	**Rel. score**	**Start**	**Strand**	**Sequence**
1	9.148	0.93881	-9246	-1	AGACGTGG
2	8.891	0.93115	-7075	1	ATACGTGA
3	9.694	0.95510	-2150	1	GTACGTGA
4	7.933	0.90257	-1072	-1	GGGCGTGT
5	8.295	0.91337	-532	-1	GGGCGTGG
6	9.817	0.95877	2510	1	GGACGTGA
7	9.542	0.95057	9670	-1	GGGCGTGC

Zebrafish 2					
**Site**	**Score**	**Rel. score**	**Start**	**Strand**	**Sequence**
1	8.575	0.92172	-9260	-1	CCGCGTGC
2	8.575	0.92172	-6523	-1	ACACGTGT
3	8.575	0.92172	-6523	1	ACACGTGT
4	8.663	0.92434	1373	-1	ATACGTGT
5	8.711	0.92578	1406	1	CTACGTGT
6	9.014	0.93482	1413	-1	AGACGTGA
7	8.295	0.91337	3945	1	GGGCGTGG
8	8.037	0.90567	4012	-1	GTGCGTGA
9	7.933	0.90257	4274	1	GGGCGTGT
10	8.295	0.91337	4732	1	GGGCGTGG
11	8.037	0.90567	4799	-1	GTGCGTGA
12	7.933	0.90257	5061	1	GGGCGTGT
13	7.933	0.90257	5368	1	GGGCGTGT
14	9.542	0.95057	6794	-1	GGGCGTGC
15	9.419	0.94690	8115	-1	GTGCGTGC
16	9.419	0.94690	8119	-1	GTGCGTGC
17	9.330	0.94424	8123	-1	GCGCGTGC
18	8.663	0.92434	9593	1	ATACGTGT

Supplementary Table 6 Primer and fragment sequences used for molecular cloning.

<table>
<tr><td colspan="2">mouse Ahsg 500bp promoter fragment</td></tr>
<tr><td colspan="2">GCTCCGGAGATTGGGAACTTGTTCCTCTCAGCAAGCTTTGCCACACTGCATGGAGATCCGTGTGCGTACAGAGC
ACTGTTACCCCCATCATGACATGAGCGCTCAGGGAAAGCGCACACAAGTCAATCAAATAACAGAGGTGCACCAC
AGGATTACACAGAGTCCTTACAGAAGTGTTTTAAGCCTTTTCAATCACATGAAGCAATATTCACATGATAGTGC
TAAATGGAAAAGGAAACAGCTACACGCTTCTATTTGGTACAGAGAAGTGAGGTGTGTGGTTAAGAGCGACTGCT
ATCTCTCCTCTCACCACGTCGCTGGGGGAGAGACAGCCAACCGCTAGCTTAAATGCCACTGTTTGTTACACTGC
TATCTTGGTTTCCACCCCCCATGTGAAATTTCATTTTGATAGCATTTAAATCTCTCCCCAGGCAGACAGGTGTA
GCACTGGGAGATGCTCCCGAGTGGCTGGCTGGCTGGCTGGCTGGCAGACC</td></tr>
<tr><td colspan="2">mouse Ahsg 500bp promoter fragment mutated</td></tr>
<tr><td colspan="2">GCTCCGGAGATTGGGAACTTGTTCCTCTCAGCAAGCTTTGCCACACTGCATGGAGATCCGTGTGCGTACAGAGC
ACTGTTACCCCCATCATGACATGAGCGCTCAGGGAAAGCGCACACAAGTCAATCAAATAACAGAGGTGCACCAC
AGGATTACACAGAGTCCTTACAGAAGTGTTTTAAGCCTTTTCAATCACATGAAGCAATATTCACATGATAGTGC
TAAATGGAAAAGGAAACAGCTACACGCTTCTATTTGGTACAGAGAAGTGAGGTGTGTGGTTAAGAGCGACTGCT
ATCTCTCCTCTCACATAATAGCTGGGGGAGAGACAGCCAACCGCTAGCTTAAATGCCACTGTTTGTTACACTGC
TATCTTGGTTTCCACCCCCCATGTGAAATTTCATTTTGATAGCATTTAAATCTCTCCCCAGGCAGACAGGTGTA
GCACTGGGAGATGCTCCCGAGTGGCTGGCTGGCTGGCTGGCTGGCAGACC</td></tr>
<tr><td colspan="2">mouse Ahsg 500bp fragment of intronic sequences</td></tr>
<tr><td colspan="2">TGCTGAAGGGAAAGCCGTGAGCGAGCACTGTGCATGTGCTACGTGCTGATTGTGAGATGCTCATTATGGGATGC
CCGAGTGGATCAAGAAGTCCTAGGACCCCACCCCGCTGGCAAACTGTTCTGTGAGGCAGCTGACTGAGCACGTG
AGTGCTGCCTTGTGGTTGGTTGGTGGGTGGGTGGGTGGGTGGGAGCTAGCAACTCAATCCTTCCAATTGCCCAG
GGCCACACGTAGTCCGTGTGGATTTGTGTTCTCTCAAACTGTGCCAGACCATAGCTTCTCCTCTCCATCCCTCC
CACCCTCCCTCTTACCTTCCTTCCTCCTCTCCTTTGCTCCCTCCCTCCCTCTCCCTCCTCCTCTCTGTCACTTC
CTCCCTCTATCCCACCTCTCCTTCCCCCCTCCCTCCCTCTGTCCCACCTCTTCTTGCCCCTCTTGTTCCAGACC
ATCTTTCCCACGTGTTCTCATTTTCATTCTCAGCCTCTCCCAGT</td></tr>
<tr><td colspan="2">mouse Ahsg 500bp fragment of intronic sequences mutated</td></tr>
<tr><td colspan="2">TGCTGAAGGGAAAGCCGTGAGCGAGCACTGTGCATGTGCATAATAATGATTGTGAGATGCTCATTATGGGATGC
CCGAGTGGATCAAGAAGTCCTAGGACCCCACCCCGCTGGCAAACTGTTCTGTGAGGCAGCTGACTGATAATAAG
AGTGCTGCCTTGTGGTTGGTTGGTGGGTGGGTGGGTGGGTGGGAGCTAGCAACTCAATCCTTCCAATTGCCCAG
GGCCAATAATAGTCCGTGTGGATTTGTGTTCTCTCAAACTGTGCCAGACCATAGCTTCTCCTCTCCATCCCTCC
CACCCTCCCTCTTACCTTCCTTCCTCCTCTCCTTTGCTCCCTCCCTCCCTCTCCCTCCTCCTCTCTGTCACTTC
CTCCCTCTATCCCACCTCTCCTTCCCCCCTCCCTCCCTCTGTCCCACCTCTTCTTGCCCCTCTTGTTCCAGACC
ATCTTTTAATAATATTCTCATTTTCATTCTCAGCCTCTCCCAGT</td></tr>
<tr><td colspan="2">mouse Ahsg 2.5kb promoter fragment</td></tr>
<tr><td>Forward Primer</td><td>atagagctccggagattgggaacttgt</td></tr>
<tr><td>Reverse Primer</td><td>aaaagaattcatggttgctccagagaggc</td></tr>
</table>

Supplementary Table 7 Primary and secondary antibodies.

Primary antibodies				
**Target/protein**	**Host species**	**Clonality**	**Application**	**Source**
Alpha smooth muscle actin	rabbit	mono	WB	Abcam, ab32575
Aquaporin-1	rabbit	poly	IF	SCBT, sc-20810
Aquaporin-2	rabbit	poly	IF	J. Loffing[18]
Beta actin	rabbit	poly	WB	Abcam, ab8227
Caspase-3 (cleaved)	rabbit	poly	IF	Cell Signaling, 9661S
CD11b-PE	rat	mono	FACS	eBioscience, 12-0112-82
CD11c-BV 605	hamster	mono	FACS	BioLegend, 117333
CD68-APC-Cy7	rat	mono	FACS	BioLegend, 137024
CD80-Pacific blue	hamster	mono	FACS	BioLegend, 104723
CD86-APC	rat	mono	FACS	BioLegend, 105011
CD206-FITC	rat	mono	FACS	BioLegend, 141703
Collagen 1	mouse	mono	WB	SCBT, sc-293182
F4/80-PE-Cy7	rat	mono	FACS	BioLegend, 123114
Fetuin-A	goat	poly	IF, WB	SCBT, sc-9668
Fetuin-A	rabbit	poly	WB	W. Jahnen-Dechent[19]
Fetuin-A	rabbit	mono	IF, IHC	Sino Biological, 50093-R022

Fibronectin	mouse	mono	WB	SCBT, sc-73611
Ncc	rabbit	poly	IF	J. Loffing[20]
Nephrin	goat	poly	IF	R&D, AF3159
Nkcc2	rabbit	poly	IF	J. Loffing[18]
Phospho-Smad3	rabbit	mono	WB	Cell Signaling, 9520S
Smad3	mouse	mono	WB	SCBT, sc-101154
Vimentin	rabbit	mono	WB	Cell Signaling, 5741T
Secondary antibodies				
**Target**	**Host species**	**Conjugate**	**Application**	**Source**
goat	donkey	Alexa Fluor® 647	IF	Jackson, 705-605-147
goat	donkey	Cyanine Cy™2	IF	Jackson, 705-225-147
goat	donkey	HRP	WB	Jackson, 705-035-147
goat	donkey	HRP	IHC	SCBT, sc-2304
mouse	goat	HRP polymer	IHC	Agilent, K4001
mouse	donkey	HRP	WB	Jackson, 715-035-151
rabbit	goat	HRP polymer	IHC	Agilent, K4003
rabbit	donkey	Cyanine Cy™3	IF	Jackson, 711-165-152
rabbit	donkey	HRP	WB	Jackson, 711-035-152
rat	donkey	Alexa Fluor® 647	IF	Jackson, 712-606-153

Supplementary Table 8 P-values for postnatal growth curve (Figure 1g).

**Postnatal day**	**No wt vs. No KO** (whites cirles)	**Hy wt vs. Hy KO** (grey cirles)	**No wt vs. Hy wt** (small asterisks)	**No KO vs. Hy KO** (large asterisks)
1	0.1171	**0.0795**	**< 0.0001**	**< 0.0001**
2	0.2810	**< 0.0001**	**0.0002**	**< 0.0001**
3	0.4101	**< 0.0001**	**0.0311**	**< 0.0001**
4	0.6130	**0.0001**	0.9948	**0.0003**
5	0.3534	**< 0.0001**	0.6691	**< 0.0001**
6	0.2508	**< 0.0001**	0.7768	**0.0004**
7	0.4692	**0.0005**	0.8715	**0.0123**
8	0.3583	**< 0.0001**	0.4804	**0.0223**
9	0.1710	**0.0020**	0.6816	0.3367
10	0.1132	**0.0014**	0.4091	0.7761
11	0.1306	**0.0027**	0.2492	0.5394
12	0.1143	**0.0040**	0.3040	0.2352
13	0.1195	**0.0020**	0.1998	0.1429
14	0.0763	**0.0023**	0.1502	**0.0368**
15	0.0918	**0.0047**	0.1509	**0.0324**
16	0.0777	**0.0103**	0.1169	**0.0059**
17	0.0897	**0.0067**	0.0567	**0.0054**
18	0.0798	**0.0067**	**0.0434**	**0.0013**
19	0.0879	**0.0032**	**0.0368**	**0.0014**
20	0.0675	**0.0024**	0.1068	**0.0069**
21	**0.0422**	**0.0003**	0.2543	0.1720
22	**0.0362**	**0.0005**	0.3287	0.2502
23	**0.0328**	**0.0010**	0.4982	0.3645
24	**0.0378**	**0.0001**	0.3984	0.4905
25	**0.0292**	**0.0001**	0.4332	0.6058
26	**0.0158**	**< 0.0001**	0.3983	0.8054
27	**0.0227**	**< 0.0001**	0.3472	0.7794
28	**0.0146**	**< 0.0001**	0.5062	0.9593
35	**0.0173**	**0.0017**	0.9430	0.7905
42	0.0748	**0.0106**	0.8946	0.8382
49	0.2117	0.118848	0.9004	0.9142
56	0.2569	0.154605	0.8423	0.9054

Supplementary References

1 Wu, C.Y., Young, L., Young, D., Martel, J. & Young, J.D. Bions: a family of biomimetic mineralo-organic complexes derived from biological fluids. *PLoS One* **8**, e75501 (2013).

2 Smith, E.R., Hewitson, T.D., Hanssen, E. & Holt, S.G. Biochemical transformation of calciprotein particles in uraemia. *Bone* **110**, 355-367 (2018).

3 Vij, A. G. Effect of prolonged stay at high altitude on platelet aggregation and fibrinogen levels. *Platelets* **20**, 421-427, doi:10.1080/09537100903116516 (2009).

4 Suresh, M. V. *et al.* Hypoxia-Inducible Factor (HIF)-1α Promotes Inflammation and Injury Following Aspiration-Induced Lung Injury in Mice. *Shock*, doi:10.1097/SHK.0000000000001312 (2018).

5 Rolfs, A., Kvietikova, I., Gassmann, M. & Wenger, R. H. Oxygen-regulated transferrin expression is mediated by hypoxia-inducible factor-1. *J Biol Chem* **272**, 20055-20062, doi:10.1074/jbc.272.32.20055 (1997).

6 Padhy, G., Sethy, N. K., Ganju, L. & Bhargava, K. Abundance of plasma antioxidant proteins confers tolerance to acute hypobaric hypoxia exposure. *High Alt Med Biol* **14**, 289-297, doi:10.1089/ham.2012.1095 (2013).

7 Tripathy, D. *et al.* Thrombin, a mediator of cerebrovascular inflammation in AD and hypoxia. *Front Aging Neurosci* **5**, 19, doi:10.3389/fnagi.2013.00019 (2013).

8 Wenger, R. H., Rolfs, A., Marti, H. H., Bauer, C. & Gassmann, M. Hypoxia, a novel inducer of acute phase gene expression in a human hepatoma cell line. *J Biol Chem* **270**, 27865-27870, doi:10.1074/jbc.270.46.27865 (1995).

9 Tan, F., Ghosh, S., Mosunjac, M., Manci, E. & Ofori-Acquah, S. F. Original Research: Diametric effects of hypoxia on pathophysiology of sickle cell disease in a murine model. *Exp Biol Med (Maywood)* **241**, 766-771, doi:10.1177/1535370216642046 (2016).

10 Zhang, B. *et al.* Comparative transcriptomic and proteomic analyses provide insights into the key genes involved in high-altitude adaptation in the Tibetan pig. *Sci Rep* **7**, 3654, doi:10.1038/s41598-017-03976-3 (2017).

11 Le Jan, S. *et al.* Characterization of the expression of the hypoxia-induced genes neuritin, TXNIP and IGFBP3 in cancer. *FEBS Lett* **580**, 3395-3400, doi:10.1016/j.febslet.2006.05.011 (2006).

12 Wellmann, S. *et al.* Oxygen-regulated expression of the RNA-binding proteins RBM3 and CIRP by a HIF-1-independent mechanism. *J Cell Sci* **117**, 1785-1794, doi:10.1242/jcs.01026 (2004).

13 Zhang, Y., Wang, H., Zhang, J., Lv, J. & Huang, Y. Positive feedback loop and synergistic effects between hypoxia-inducible factor-2α and stearoyl-CoA desaturase-1 promote tumorigenesis in clear cell renal cell carcinoma. *Cancer Sci* **104**, 416-422, doi:10.1111/cas.12108 (2013).

14 Hernández, C. *et al.* Induction of trefoil factor (TFF)1, TFF2 and TFF3 by hypoxia is mediated by hypoxia inducible factor-1: implications for gastric mucosal healing. *Br J Pharmacol* **156**, 262-272, doi:10.1111/j.1476-5381.2008.00044.x (2009).

15 Liangos, O. *et al.* Whole blood transcriptomics in cardiac surgery identifies a gene regulatory network connecting ischemia reperfusion with systemic inflammation. *PLoS One* **5**, e13658, doi:10.1371/journal.pone.0013658 (2010).

16 Saraf, S. L. *et al.* Progressive glomerular and tubular damage in sickle cell trait and sickle cell anemia mouse models. *Transl Res* **197**, 1-11, doi:10.1016/j.trsl.2018.01.007 (2018).

17 Kelly, K. J., Liu, Y., Zhang, J. & Dominguez, J. H. Renal C3 complement component: feed forward to diabetic kidney disease. *Am J Nephrol* **41**, 48-56, doi:10.1159/000371426 (2015).
18 Wagner, C. A. *et al.* Mouse model of type II Bartter's syndrome. II. Altered expression of renal sodium- and water-transporting proteins. *Am J Physiol Renal Physiol* **294**, F1373-1380, doi:10.1152/ajprenal.00613.2007 (2008).
19 Denecke, B. *et al.* Tissue distribution and activity testing suggest a similar but not identical function of fetuin-B and fetuin-A. *Biochem J* **376**, 135-145, doi:10.1042/BJ20030676 (2003).
20 Loffing, J. *et al.* Altered renal distal tubule structure and renal Na(+) and Ca(2+) handling in a mouse model for Gitelman's syndrome. *J Am Soc Nephrol* **15**, 2276-2288, doi:10.1097/01.ASN.0000138234.18569.63 (2004).
