# Supplementary material for: Fetuin-A is a HIF target that safeguards tissue integrity during hypoxic stress
Source: Nat Commun. 2021 Jan 22;12:549. doi: 10.1038/s41467-020-20832-7 (PMC7822914; doi:10.1038/s41467-020-20832-7)
Supplement: Supplementary file 3 — Description of Additional Supplementary Files [file 41467_2020_20832_MOESM3_ESM.pdf]

Description of Additional Supplementary Files

File Name: Supplementary Movie 1
Description: Z-stacks of a normoxic E18.5 kidney iDisco stained for nephrin.

File Name: Supplementary Movie 2
Description: Z-stacks of a hypoxic E18.5 kidney iDisco stained for nephrin.
