# Supplementary material for: Fetuin-A is a HIF target that safeguards tissue integrity during hypoxic stress
Source: Nat Commun. 2021 Jan 22;12:549. doi: 10.1038/s41467-020-20832-7 (PMC7822914; doi:10.1038/s41467-020-20832-7)
Supplement: Supplementary file 6 — Reporting Summary [file 41467_2020_20832_MOESM6_ESM.pdf]

nature research

Corresponding author(s): Uyen Huynh-Do

Last updated by author(s): Nov 12, 2020

Reporting Summary

Nature Research wishes to improve the reproducibility of the work that we publish. This form provides structure for consistency and transparency in reporting. For further information on Nature Research policies, see Authors & Referees and the Editorial Policy Checklist.

Statistics

For all statistical analyses, confirm that the following items are present in the figure legend, table legend, main text, or Methods section.

n/a	Confirmed	
☐	☒	The exact sample size (*n*) for each experimental group/condition, given as a discrete number and unit of measurement
☐	☒	A statement on whether measurements were taken from distinct samples or whether the same sample was measured repeatedly
☐	☒	The statistical test(s) used AND whether they are one- or two-sided<br>*Only common tests should be described solely by name; describe more complex techniques in the Methods section.*
☒	☐	A description of all covariates tested
☐	☒	A description of any assumptions or corrections, such as tests of normality and adjustment for multiple comparisons
☐	☒	A full description of the statistical parameters including central tendency (e.g. means) or other basic estimates (e.g. regression coefficient) AND variation (e.g. standard deviation) or associated estimates of uncertainty (e.g. confidence intervals)
☐	☒	For null hypothesis testing, the test statistic (e.g. *F*, *t*, *r*) with confidence intervals, effect sizes, degrees of freedom and *P* value noted<br>*Give P values as exact values whenever suitable.*
☒	☐	For Bayesian analysis, information on the choice of priors and Markov chain Monte Carlo settings
☒	☐	For hierarchical and complex designs, identification of the appropriate level for tests and full reporting of outcomes
☒	☐	Estimates of effect sizes (e.g. Cohen's *d*, Pearson's *r*), indicating how they were calculated

Our web collection on statistics for biologists contains articles on many of the points above.

Software and code

Policy information about availability of computer code

Data collection	Protein and Creatinine concentration, ELISA: XFluor4, V4.51 in Microsoft Excel. Imaging: Live Acquisition software (FEI, V2.6.0.14); Nikon NIS Elements 4.0; Leica Acquire V3.4.5. FACS: FACSDiva and FlowJo V10.6.1. Microarray: Affymetrix Power Tools software package using Affymetrix Expression Console software (V1.1.2800.28061). RT-qPCR: 7500 Fast System SDS, V1.4 (Applied Biosystems). Luciferase: Fluoroskan Ascent Software, V2.6. ELISA: XFluor4, V4.51 in Microsoft Excel.
Data analysis	GFR: MPD lab, V1.0a. Glomerular count: Fiji, ImageJ version 2.0.0-rc69/1.52i (https://imagej.net) and TrackMate V3.8.0 plugin. Imaging: Offline Analysis software (FEI, V2.6.0.14); Adobe Photoshop CC V19.0; Adobe Illustrator CC V22.0.1. FACS: FACSDiva and FlowJo V10.6.1. Microarray: Affymetrix Power Tools software package using Affymetrix Expression Console software (V1.1.2800.28061), Robust Multi-array Average algorithm (RMA) and R Bioconductor package “limma”; Heatmapper (online tool at www.heatmapper.ca); DAVID Bioinformatics Resources V6.8 (https://david.ncifcrf.gov). RT-qPCR: Microsoft Excel for Mac V16.43. HIF binding sites: The Galaxy Project (online tool at https://usegalaxy.org); Microsoft Excel for Mac V16.43. Molecular Cloning: DNAstar Lasergene V13.0; SnapGene Viewer V5.1.3. Luciferase: Fluoroskan Ascent Software, V2.6. ELISA: Microsoft Excel for Mac V16.43. Western blot: Adobe Photoshop CC V19.0; Adobe Illustrator CC V22.0.1; Microsoft Excel for Mac V16.43. Data analysis and graphical design: Adobe Photoshop CC V19.0; Adobe Illustrator CC V22.0.1; Prism 8 for MacOS V8.4.3.

For manuscripts utilizing custom algorithms or software that are central to the research but not yet described in published literature, software must be made available to editors/reviewers. We strongly encourage code deposition in a community repository (e.g. GitHub). See the Nature Research guidelines for submitting code & software for further information.

Data

Policy information about availability of data

All manuscripts must include a data availability statement. This statement should provide the following information, where applicable:

- Accession codes, unique identifiers, or web links for publicly available datasets
- A list of figures that have associated raw data
- A description of any restrictions on data availability

Microarray raw data were deposited at the GEO genomics data repository under the accession code GSE148778 [https://www.ncbi.nlm.nih.gov/geo/query/acc.cgi?acc=GSE148778]. HIF ChIP-seq datasets are accessible under GSE28352 [https://www.ncbi.nlm.nih.gov/geo/query/acc.cgi?acc=gse28352]. Other genome wide dataset were obtained from UCSC Genome Browser at https://genome.ucsc.edu (for human: H3K27Ac, H3K4Me1, DNaseI HS (ENCODE); for mouse DNaseI HS (ENCODE/UW),H3K27Ac and H3K4Me1 (ENCODE/LICR)) or JASPAR database at http://jaspar.genereg.net (for potential mouse HIF binding sites). The source data underlying Figs. 1b-g, 2, 4c-d, 5a-e and j, 6, 7e-f and k, 8l-m and Supplementary Figs. 1, 3, and 6-9 are provided as a Source Data file. All other data supporting the findings of this study are available from the corresponding author on request.

Field-specific reporting

Please select the one below that is the best fit for your research. If you are not sure, read the appropriate sections before making your selection.

☒ Life sciences ☐ Behavioural & social sciences ☐ Ecological, evolutionary & environmental sciences

For a reference copy of the document with all sections, see nature.com/documents/nr-reporting-summary-flat.pdf

Life sciences study design

All studies must disclose on these points even when the disclosure is negative.

Sample size	Our study is an explorative study based on the results of a gene expression analysis between normoxic and hypoxic fetal kidneys. For the microarray, 3 mice per group were used to minimize costs and to obtain a data set sufficiently large to perform statistical analysis. Fetuin-A was the gene showing the highest induction in hypoxic kidneys and caught our undivided attention, given its prognostic importance for the progression of chronic kidney disease in humans. In the wake of this initial finding our goal was to investigate the mechanisms underlying the ectopic induction of this protein in the kidney and to clarify its role in hypoxia-induced IUGR and kidney development. Thus, a power analysis to pre-determine the sample size for our studies was not adequately possible. We decided to use a manageable amount of animals to examine the open questions. Detailed numbers on sample size are provided in the figures or figure legend.<br><br>Retrospectively, the number of animals per groups was not arbitrarily chosen, but rather matched the minimum sample size requirements to detect differences with an alpha error = 0.05 and a beta error = 0.2, corresponding to a power of 80 %. For example, in our GFR analysis the variability of each group was around 10 % and the smallest difference between the groups was approximately 15 %, which in a power analysis results in a sample size of 7 animals per group. Similarly, for the proteinuria measurements the variability of each group was around 15 % and the smallest difference between the groups was approximately 25 %, which in a power analysis results in a sample size of 6 animals per group.
Data exclusions	No data was excluded.
Replication	Our data is based on at least 2 to 3 independent experiments. All replications were successful. No experimental finding could not be replicated.
Randomization	Animals were randomly divided into experimental groups.
Blinding	Blinding was not used in this study, as the experimental conditions (hypoxia, normoxia, Cc) were evident.

Reporting for specific materials, systems and methods

We require information from authors about some types of materials, experimental systems and methods used in many studies. Here, indicate whether each material, system or method listed is relevant to your study. If you are not sure if a list item applies to your research, read the appropriate section before selecting a response.

Materials & experimental systems

n/a	Involved in the study	
☐	☒	Antibodies
☐	☒	Eukaryotic cell lines
☒	☐	Palaeontology
☐	☒	Animals and other organisms
☒	☐	Human research participants
☒	☐	Clinical data

Methods

n/a	Involved in the study	
☒	☐	ChIP-seq
☐	☒	Flow cytometry
☒	☐	MRI-based neuroimaging

Antibodies

Antibodies used	Primary antibodies<br>Alpha smooth muscle actin, Abcam, ab32575; Aquaporin-1, SCBT, sc-20810; Aquaporin-2, Prof. Loffing, Zurich (Wagner et al., 2008); Beta actin, Abcam, ab8227; Caspase-3 (cleaved), Cell Signaling, 9661S; Collagen 1, SCBT, sc-293182; Fetuin-A, SCBT, sc-9668; Fetuin-A, Prof. Jahnen-Dechent (Denecke et al., 2003); Fetuin-A, Sino Biological, 50093-R022; Fibronectin, SCBT, sc-73611; Ncc, Prof. Loffing (Loffing et al., 2004); Nephrin, R&D, AF3159; Nkcc2, Prof. Loffing (Wagner et al., 2008); Phospho-Smad3, Cell Signaling, 9520S; Smad3, SCBT, sc-101154; Vimentin, Cell Signaling, 5741T; CD11b-PE, eBioscience, 12-0112-82; CD11c-BV 605, BioLegend, 117333; CD68-APC-Cy7, BioLegend, 137024; CD80-Pacific blue, BioLegend, 104723; CD86-APC, BioLegend, 105011; CD206-FITC, BioLegend, 141703; F4/80-PE-Cy7, BioLegend, 123114.<br><br>Secondary antibodies<br>goat Alexa Fluor® 647, Jackson, 705-605-147; goat Cyanine Cy™2, Jackson, 705-225-147; goat HRP, Jackson, 705-035-147; goat HRP, SCBT, sc-2304; mouse HRP polymer, Agilent, K4001; rabbit HRP polymer, Agilent, K4003; mouse HRP, Jackson, 715-035-151; rabbit Cyanine Cy™3, Jackson, 711-165-152; rabbit HRP, Jackson, 711-035-152; rat Alexa Fluor® 647, Jackson, 712-606-153.
Validation	All antibodies were used according to the profile of manufacturer. Fetuin-A antibodies were validated for WB, IF and IHC using KO animals. Commercial antibodies were validated by the manufacturer: Alpha smooth muscle actin, Abcam, ab32575: validated for mouse WB (NIH/3T3 cells); Aquaporin-1, SCBT, sc-20810: validated for mouse IHC (mouse kidney), not validated, but recommended for mouse IF; Beta actin, Abcam, ab8227: validated for mouse WB (NIH/3T3 cells); Caspase-3 (cleaved), Cell Signaling, 9661S: validated for human IF (HT-29 cells), not validated, but recommeded for mouse IF; CD11b-PE, eBioscience, 12-0112-82: validated for mouse FACS (mouse bone marrow cells); CD11c-BV 605, BioLegend, 117333: validated for mouse FACS (C57BL/6 splenocytes); CD68-APC-Cy7, BioLegend, 137024: validated for mouse FACS (BALB/c macrophages); CD80-Pacific, BioLegend, 104723: validated for mouse FACS (C57BL/6 splenocytes); CD86-APC, BioLegend, 105011: validated for mouse FACS (C57BL/6 splenocytes); CD206-FITC, FACS BioLegend, 141703: validated for mouse FACS (BALB/c macrophages); Collagen 1, SCBT, sc-293182: validated for human WB (732.Sk/Mu cells), not validated, but recommended for mouse WB; F4/80-PE-Cy7, BioLegend, 123114: validated for mouse FACS (BALB/c peritoneal macrophages); Fetuin-A, SCBT, sc-9668: validated for mouse WB (mouse liver tissue extract), not validated, but recommeded for mouse IF; Fetuin-A, Sino Biological, 50093-R022: validated for mouse IHC (mouse liver), not validated for mouse IF; Fibronectin, SCBT, sc-73611: validated for human WB (HT-1080 cells), not validated for mouse WB; Nephrin, R&D, AF3159: validated for mouse IF (mouse kidney); Phospho-Smad3, Cell Signaling, 9520S: validated for mouse WB (C2C12 cells); Smad3, SCBT, sc-101154: validated for human WB (293T cells), not validated, but recommended for mouse WB; Vimentin, Cell Signaling, 5741T: validated for mouse WB (NIH/3T3 cells).<br>Home-made antibodies were validated by the providing scientist as stated in their original publications: Aquaporin-2, Wagner et al., 2008: validated for mouse IF (mouse kidney); Fetuin-A, Denecke et al., 2003: validated for mouse WB (mouse liver); Ncc, Loffing et al., 2004: validated for mouse IF (mouse kidney); Nkcc2 Wagner et al., 2008: validated for mouse IF (mouse kidney).<br>For Aquaporuin-2, Nkcc2, specific binding was inhibited by preincubation with the specific peptides used for immunization.<br>Fetuin-A and Ncc antibodies were validated using KO animals.

Eukaryotic cell lines

Policy information about cell lines

Cell line source(s)	Primary cells of the proximal tubulus (pPTCs) were freshly isolated before each experiment. NRK cells and HK-2 cells were obtained from ATCC.
Authentication	NRK cells and HK-2 were assessed for the expression of renal genes using a panel of genes representing different kidney segments.
Mycoplasma contamination	All cell lines tested negative for mycoplasma contamination.
Commonly misidentified lines<br>(See ICLAC register)	No such lines were used.

Animals and other organisms

Policy information about studies involving animals; ARRIVE guidelines recommended for reporting animal research

Laboratory animals	All mice, including Ahsgtm1Mbl mice and Clcn5tm1Gug mice (kindly provided by Prof. Olivier Devuyst, University of Zurich) were maintained on a C57BL/6 background. All mice were housed in IVC cages with free access to chow and water and a 12h day/night cycle, 23 °C ambient temperature and 40-60% humidity.
Wild animals	The study did not involve wild animals.
Field-collected samples	The study did not involve field-collected samples.
Ethics oversight	Breeding, genotyping and all animal experiments were conducted according to the Swiss law for the welfare of animals and were approved by the local authorities (Canton of Bern - permissions BE96/11, BE105/14 and BE105/17).

Note that full information on the approval of the study protocol must also be provided in the manuscript.

Flow Cytometry

Plots

Confirm that:

☒ The axis labels state the marker and fluorochrome used (e.g. CD4-FITC).

☒ The axis scales are clearly visible. Include numbers along axes only for bottom left plot of group (a 'group' is an analysis of identical markers).

☒ All plots are contour plots with outliers or pseudocolor plots.

☒ A numerical value for number of cells or percentage (with statistics) is provided.

Methodology

Sample preparation	Fetal kidneys were dissociated with collagenase I (2mg/ml in 1% BSA/PBS; C9891, Sigma) for 20 min at 37°C. The cell suspension was was filtered through a 40 µm pore size filter, washed and then stained using a panel of antibodies for 30 min at 4°C. Antibodies are provided in Supplementary Table 7.
Instrument	Cells were sorted on a SORP LSRII
Software	FACSDiva and analysed with FlowJo 10.6.1.
Cell population abundance	Whole fetal kidneys were sorted for macrophages without a purification of cells. The abundance of macrophages in fetal kidneys was approximately 2.5%.
Gating strategy	Due to the heterogeneity of the cell suspension (comprising stem cells, epithelial cells, endothelial cells and immune cells) they were first gated for CD11b/SSC-A. CD11b is a pan macrophage marker. Then single cells were selected. The single cells were further gated for CD11b and F4/80 to differentiate resident and infiltrating macrophage populations. These populations were then gated for various M1 markers and CD206. Alternatively, the single cells were gated for F4/80 and CD206 to distinguished M1 and M2 polarized cells.

☒ Tick this box to confirm that a figure exemplifying the gating strategy is provided in the Supplementary Information.
